# Global Plant Virus Disease Pandemics and Epidemics

**DOI:** 10.3390/plants10020233

**Published:** 2021-01-25

**Authors:** Roger A. C. Jones

**Affiliations:** The UWA Institute of Agriculture, University of Western Australia, 35 Stirling Highway, Crawley, WA 6009, Australia; roger.jones@uwa.edu.au

**Keywords:** pandemics, epidemics, global, virus, disease, crop losses, crop failure, food insecurity, threat, devastation, developing countries, domestication centers, dissemination, evolution, international trade, germplasm distribution, integrated disease management

## Abstract

The world’s staple food crops, and other food crops that optimize human nutrition, suffer from global virus disease pandemics and epidemics that greatly diminish their yields and/or produce quality. This situation is becoming increasingly serious because of the human population’s growing food requirements and increasing difficulties in managing virus diseases effectively arising from global warming. This review provides historical and recent information about virus disease pandemics and major epidemics that originated within different world regions, spread to other continents, and now have very wide distributions. Because they threaten food security, all are cause for considerable concern for humanity. The pandemic disease examples described are six (maize lethal necrosis, rice tungro, sweet potato virus, banana bunchy top, citrus tristeza, plum pox). The major epidemic disease examples described are seven (wheat yellow dwarf, wheat streak mosaic, potato tuber necrotic ringspot, faba bean necrotic yellows, pepino mosaic, tomato brown rugose fruit, and cucumber green mottle mosaic). Most examples involve long-distance virus dispersal, albeit inadvertent, by international trade in seed or planting material. With every example, the factors responsible for its development, geographical distribution and global importance are explained. Finally, an overall explanation is given of how to manage global virus disease pandemics and epidemics effectively.

## 1. Introduction

Virus disease pandemics and major epidemics threaten cultivated plants grown not only to feed humankind and its livestock, but also to produce fiber, ornamental plant or medicinal products. When many plants become systemically virus-infected and this infection causes severe disease symptoms, the magnitude of the resulting losses in overall crop yield or quality of produce can be devastating. Moreover, such losses happen in both annual and perennial cultivated plants [1,2,3,4,5,6,7,8,9]. Furthermore, when virus disease pandemics or major epidemics occur in staple food crops essential for food security, they are capable of decreasing food supplies so much that severe food shortages cause famine [4,7,9,10,11,12,13,14,15,16]. In 2014, virus disease pandemics and epidemics were estimated to have a global economic impact of >US$30 billion annually [17]. Their current economic impact in terms of US$ has escalated considerably since then due to the increased scale of global agriculture and demand for plant products needed to feed the rapidly expanding human population. In addition, nearly half (47%) of the pathogens that cause emerging and re-emerging plant disease epidemics worldwide are viruses [5]. This too contributes towards amplifying the global economic impact of plant virus disease [9]. 

In global agriculture, once the cultivation of annual crops as monocultures became widespread, this practice provided an underlying basic ingredient of instability [18]. This instability increased further as a direct result of improved cultural practices and plant breeding approaches being implemented to enhance yields, expand cropping regions and extend growing seasons [4,18]. In consequence, serious virus disease epidemics became regularly recurring features in many herbaceous crops, such as the epidemic examples of cereal yellow dwarf, wheat streak mosaic, rice tungro and sugar beet yellows described by Thresh in 1980 [4]. In perennial woody crops, however, damaging virus disease epidemics occur less often because establishing virus infection tends to be more difficult than with herbaceous crops, systemic invasion is slower, and, overall, they are more likely to be tolerant or develop inconspicuous symptoms associated with minor effects on yield and produce quality [18]. Nevertheless, exceptions occur—among the most damaging being virus disease epidemics of citrus tristeza, plum pox and cocoa swollen shoot [4,18]. 

Several factors have contributed to the currently rapidly deteriorating global plant virus disease situation. Firstly, the rapid expansion of international trade in plants and plant produce by multinational companies is introducing damaging virus diseases to parts of the world where they were formerly absent. This is occurring for three main reasons. (i) Trade globalization involving international agreements over free trade or tariff reductions has opened up new pathways for the large-sale transfer of crop produce from one continent or distant country to another. (ii) Lower subsidies for developed country production have enabled developing countries to expand their trade in international crop produce. (iii) More efficient methods of rapid transport by air and sea, combined with the loosening of plant quarantine regulations to meet revised World Trade Organization rules, have facilitated this trade [7,9,19,20,21]. Secondly, damaging new virus diseases are emerging at an accelerating rate due to the movement of crop plants away from their domestication centers to distant countries or continents where they are cultivated as monocultures. These introduced crops often become invaded by damaging viruses they never encountered previously, spreading to them from natural vegetation [4,7,13,14,18,22,23,24,25]. Thirdly, plant virus disease pandemics and epidemics are becoming increasingly difficult to manage due to climate instability arising from global warming [7,16,26,27,28,29]. An important example of (i) is the tendency of multinational seed companies to produce seed crops in distant developing countries with warmer climates at times of year when they cannot be grown in regions with temperate climates. The downside of this is that it risks seed crop infection by previously unknown seed-borne viruses spreading into them from local indigenous crops or natural vegetation followed by their inadvertent worldwide distribution by the seed trade [7,16,19,30,31].

Reviews and research papers by the late J.M. Thresh [32] described plant virus disease pandemics or major epidemics that occurred across the world between the early 1900s and 2006, e.g., [4,6,12,13,14,18,22,33,34,35,36,37]. His comprehensive global review published in 1980 described the origins and epidemiology of important plant virus diseases up until then [4]. In 1999, Rybicki and Pietersen [38] reviewed plant virus diseases in developing countries but focused mostly on describing the causal viruses rather than the diseases that they engendered. More recently, several reviews described virus disease pandemics or major epidemics involving single pathosystems [39,40,41,42,43,44,45]. In 2019, a general review addressed the global dimensions of plant virus disease [9]. In 2020, Jones [25] reviewed historical and recent information concerning virus disease pandemics and major epidemics arising from new encounters between indigenous viruses and crops introduced from another continent. The principal virus disease examples described were: (i) rice yellow mottle, cassava brown streak, groundnut rosette and cocoa swollen shoot, which are limited to sub-Saharan Africa (SSA); (ii) cassava mosaic in SSA, the Indian subcontinent and Southeast Asia; and (iii) tomato yellow leaf curl, which to all extents and purposes now has a global distribution. 

This review focusses mainly on virus disease pandemics or major epidemics that are known to have, or considered likely to have, originated within crop domestication centers in different parts of the world, became distributed from there to other continents, and now have predominantly global distributions. The examples described infect cereals (maize, rice and wheat), root and tuber crops (potato and sweet potato), plantation and orchard crops (banana, citrus and stone fruit), grain legumes (faba bean) and annual horticultural crops (tomato and cucurbits). Both historical and recent information is provided about seven examples of virus diseases that threaten staple food crops and therefore are of critical importance regarding global food security. Three of these seven staple food crop examples involve diseases caused by virus complexes. The remaining six examples involve virus diseases threatening other food crops that play important roles in achieving balanced human nutrition. Three of the latter examples are of seed-borne virus diseases currently of concern because of their inadvertent dispersal by the international seed trade. The ways each of these 13 examples arose, and then became distributed globally, is described, and information is given about the principal factors favoring their development. Lastly, a brief overall summary is provided concerning how to achieve successful management of global virus disease pandemics and major epidemics that afflict important food crops.

## 2. Definitions and Concepts

### 2.1. Definitions

In his 1973 review of plant diseases threatening worldwide agriculture, Thurston [2] categorized them as ‘highly threatening’, or of ‘intermediate threat’ or ‘limited threat potential’. A disease was categorized as ‘highly threatening’ when it spread rapidly, caused serious losses and was difficult to control, whereas those of ‘limited threat potential’ spread slowly and/or were easy to control. The six virus (or viroid) disease examples he included were African cassava mosaic (highly threatening), banana bunchy top, maize streak, pangola grass stunt, cocoa swollen shoot (intermediate threat) and coconut cadang cadang (limited threat).

When he reviewed catastrophic plant diseases in 1970, Klinkowski [1] described how plant disease agents, including viruses, cause both epidemics and pandemics. He defined an ‘epidemic’ as being “where a disease is spread over an area in which its causal agent has been present for a long time”; and a ‘pandemic’ as occurring “where epidemics cause mass infections spread over several continents”. More recently, for plant virologists, an ‘epidemic’ has come to mean “a marked increase in the incidence of virus disease within a plant population”. This definition includes situations not only where the diseased plant population is localized, e.g., to a single field, but also where it occurs over a very wide area. In addition, a ‘pandemic’ has become “an epidemic occurring over a very wide area, crossing international boundaries and causing severe crop losses consistently”. Notably, this ‘pandemic’ definition includes situations when the severely affected countries occur in different continents [1], and when they are all within in the same continent, e.g., in SSA [36,37,45]. Jones [25] used the additional term ‘major epidemic’ to mean “an epidemic occurring over a wide area, crossing international boundaries and causing severe crop losses sporadically”. Thus, although the losses are severe and develop over a wide area in this latter category, the epidemics causing them occur sporadically so use of the ‘pandemic’ is unwarranted. 

To understand plant virus epidemics and pandemics, it is also necessary to understand what plant virologists mean when they refer to the term ‘plant virus disease epidemiology’. Various definitions have been suggested, including the study of: (i) “the patterns of disease within space and time, and within populations” [46]; (ii) “the cyclical development of virus diseases within plant populations in time and space” [47]; and (iii) “the determinants, dynamics and distribution of virus diseases within host populations” [8]. ‘Plant virus disease epidemiology’ has also been defined as “the complex association between a virus and its host plant resulting in disease, and the factors that influence spread within the host plant population” [48]. Therefore, epidemiology concerns how and why a virus spreads in a plant population, and the consequences in terms of disease [16]. For the effective management of a plant virus disease, first obtaining an understanding of its epidemiology is essential [9,13,14,22,23,49,50]. Furthermore, when considering virus disease management, it is important to clarify the terms that plant virologists use to categorize different types of host responses to virus infection in crop cultivars. The propensity of ‘vulnerable’ crop cultivars to develop systemic virus infection resulting in severe systemic disease symptoms, constitutes a major determinant when it comes to the magnitude of the yield and quality losses incurred [14,18]. Thus, being ‘vulnerable’ means that “the cultivar is both ‘susceptible’ to virus infection (i.e., it becomes infected readily), and ‘sensitive’ to infection once systemic infection has occurred (i.e., it develops severe symptoms)”. Having ‘resistance’ to virus infection is the opposite of being ‘susceptible’ and having ‘tolerance’ is the opposite of being ‘sensitive’ [4,14,51]. 

### 2.2. Concepts

Some pandemics or major virus disease epidemics result from new encounter scenarios when a crop first domesticated in one continent [52,53] is introduced to another continent where it becomes infected by an indigenous virus it has not met previously [4,7,13,25,54,55]. Moreover, the country or region where the virus responsible for causing a pandemic or epidemic was initially found does not necessarily mean that this is where this virus first originated. In many cases, it is likely to have coevolved with the crop in its crop domestication center having already been present infecting the crop’s wild ancestor(s) or having spread to it from nearby wild plants. Infected seeds or vegetatively propagated planting material may then have been taken somewhere else and planted in the place where the causal virus was identified first. Establishing whether the causal agent of a virus disease pandemic or epidemic first arose in a crop domestication center requires a thorough investigation of the nucleotide sequence diversity occurring amongst virus isolates from there. When viruses coevolve for a very long period locally within the same plant species, a high degree of nucleotide sequence diversity occurs amongst isolates collected over a small geographic range [7,56]. For example, such diversity was evident when isolates of potato virus A (genus *Potyvirus*, family *Potyviridae*), potato virus S (genus, *Carlavirus*, family *Betaflexiviridae*) and potato virus Y (PVY, genus *Potyvirus*, family *Potyviridae*) were collected from plantings in potato’s Andean crop domestication center [57,58,59]. Unfortunately, phylogenetic studies with enough causal virus sequences from crop domestication centers were only available to draw conclusions with four of the 13 global virus disease pandemics or epidemics described in this review. These viruses are PVY [58]; plum pox virus (PPV; genus *Potyvirus*, family *Potyviridae*) [60]; rice tungro bacilliform virus (RTBV; genus, *Tungrovirus,* family, *Caulimoviridae*) and rice tungro spherical virus (RTSV; genus, *Waikavirus*, family, *Secoviridae*) [61,62]; and banana bunchy top virus (BBTV; genus, *Babuvirus*, family, *Nanoviridae*) [63,64]. 

## 3. Cereals

### 3.1. Maize

Overall, maize (*Zea mays*) is now the world’s most important staple food crop, and the second most important in the developing world [65,66]. It was domesticated in Western Mexico from its wild ancestor *Zea mays* ssp. *parviglumis* [67]. In the pre-Columbian era, it was dispersed widely through Central and South America, and the Caribbean [68]. After the Spanish arrived in the Americas in 1492, they introduced maize to Europe after which it was taken from there to other continents. Maize was introduced to Africa in the early 17th century, where its high yields and short growing season favored its rapid adoption [69]. Maize crops suffer from many virus diseases [70]. An example of a devastating maize virus disease pandemic is described below.

#### Maize Lethal Necrosis Disease

**Figure 1:**
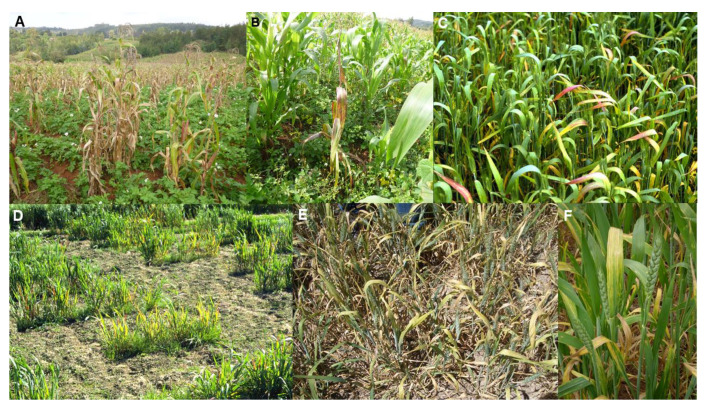
(**A**) East African smallholder maize crop devastated by maize lethal necrosis disease (all maize plants killed or dying); potato understory crop unaffected. (**B**) Maize plant dying from maize lethal necrosis disease (center), surrounding maize plants healthy, image modified from [9]. (**C**) Wheat crop containing yellow dwarf diseased plants showing flag leaf symptoms of reddening and chlorosis. (**D**) Rows of wheat plants showing severe yellow dwarf disease symptoms of leaf chlorosis and reddening and plant stunting. (**E**) Wheat crop devastated by wheat streak mosaic disease showing plants with severe leaf chlorosis, deformation and necrosis, and overall plant stunting. (**F**) Close up of wheat plant showing wheat streak mosaic disease leaf symptoms of chlorotic yellowing and green streaking.

Maize lethal necrosis disease (MLND) was reported initially in 1977 in the USA, where it was called corn lethal necrosis disease [71]. Its dramatic symptoms consist of severe systemic leaf and shoot necrosis, and plant death (Figure 1A,B). The grain yields from symptomatic plants are either negligible or greatly diminished [Table 1]. Around 2010, MLND emerged causing a pandemic of maize crops in SSA, particularly those of smallholder farmers. This devastating pandemic now spans eight different East and Central African countries (Democratic Republic of Congo, Ethiopia, Kenya, Mozambique, Ruanda, South Sudan, Tanzania, and Uganda), where it damages maize crops over almost 1.2 million km^2^ [42,72]. In East and Central Africa, maize is the staple food crop of over 300 million people. MLND is now so damaging in these countries that it constitutes a major threat not only to the livelihoods of smallholder farmers that grow maize but also to overall maize production, seriously threatening food security. In addition, around 2010, MLND also appeared in East and Southeast Asia, Europe (Spain) and South America (Ecuador), the resulting grain yield losses being considerable in China, Taiwan and Ecuador [42,73]. Moreover, in the future, the likelihood of MLND causing major losses in more countries where maize is cultivated seems very high. 

MLND is caused by mixed infection between maize chlorotic mottle virus (MCMV; genus, *Machlomovirus*, family, *Tombusviridae*) and one or other of several different cereal viruses belonging to the *Potyviridae*, such as sugarcane mosaic virus (SCMV; genus, *Potyvirus*, family, *Potyviridae*), maize dwarf mosaic virus (MDMV; genus *Potyvirus*, family, *Potyviridae*) and wheat streak mosaic virus (WSMV; genus, *Tritimovirus*, family, *Potyviridae*) [42]. The two viruses interact synergistically in mixed infection causing the very severe foliage disease symptoms that culminate in MLND. Since the cereal *Potyviridae* that infect maize, especially SCMV, commonly occur in warmer climates globally, whether MLND occurs in a region depends on the presence of MCMV [42]. MCMV was found first in 1973 in Peru [137], and in the 1970s–1990s it spread to Argentina, Mexico and the USA in the Americas, and to Thailand in Southeast Asia. Since approximately 2010, it spread to Ecuador, Spain, China and Taiwan, and to several countries in SSA [42,72]. However, this sequence of events may be illusory as it might actually have co-evolved with maize in its Mexican domestication center and been dispersed elsewhere in maize as seed-borne MCMV infection, the historical sequence of its detection in different countries actually reflecting presence of virologists interested in maize diseases. MDMV likely also coevolved with Maize in Mexico, and SCMV within sugar cane’s Indian subcontinent or Southeast Asian domestication center, but this lacks confirmation by phylogeographic or other evolutionary studies. WSMV probably originated in wheat’s Middle East domestication center (see Section 3.2.2 below). Ancestral viruses might initially have spread to these crops from infected wild ancestors of maize (MCMV, MDMV), sugar cane (SCMV) or wheat (WSMV), but this too lacks confirmation.

MCMV has very stable virions, and is transmitted by contact, contaminated maize seed and soil, and several beetle and thrips species act as its vectors. Alternative hosts for MCMV include sugar cane (*Saccharum officinarum*), sorghum (*Sorghum bicolor*), millet (*Panicum miliaceum*), wheat (*Triticum aestivum*), barley (*Hordeum vulgare*), and several pasture and weed grasses. Cereal infecting *Potyviridae* are transmitted by aphids (e.g., MDMV, SCMV) or eriophyid mites (WSMV). They infect a diverse array of alternative hosts amongst poaceous crops, weeds and pasture plants [42,73]. Distribution of MCMV-contaminated maize seed by the international seed trade seems responsible for MCMV’s rapid increase in global distribution [42,73]. Moreover, along with spread of locally occurring cereal *Potyviridae* to maize plants, sowing MCMV-contaminated maize seed seems responsible for MLND’s recent emergence in SSA, and other continents outside the Americas [42,73]. MCMV isolates from East Africa most resembled MCMV isolates from China [74]. Since isolates from both regions showed little sequence diversity, its recent introduction via MCMV-contaminated maize seed from China seems likely. Because MDMV and WSMV are seed-borne in maize and wheat, respectively, most likely these two viruses were originally dispersed between continents via contaminated seeds [72] (see Section 3.2.2 below), whereas SCMV, which is not-seed-borne in sugar cane, probably originally became dispersed in infected sugar cane planting material [72]. 

What is responsible for the devastating MLND pandemic that emerged after MCMV’s arrival in SSA? Factors exacerbating MLND’s appearance include sowing vulnerable maize cultivars, agricultural intensification including sowing multiple maize crops annually, and the widespread occurrence not only of its maize thrips vector (*Frankliniella williamsi*) but also of SCMV and other cereal potyviruses infecting maize and other poaceous hosts [42]. Widespread sowing MCMV-contaminated maize seed stocks and planting the crop in virus-contaminated soils are also likely to have played important roles [73,74].

### 3.2. Wheat

Wheat was domesticated approximately 10,000 years ago in the fertile crescent region of the Middle East [138]. Its seeds were distributed from there to other parts of Asia, and then to Europe, Africa and the Americas, and, most recently, to Australasia. Worldwide, wheat crops now occupy a greater area of agricultural land than any other food crop, and this crop ranks second after maize as the most important staple food crop [66,139]. Although it is typically grown in temperate and Mediterranean-type climates, or at cooler high altitudes in warmer climates, it still plays a critical role in helping provide food security in developing countries. Wheat crops are afflicted by many virus diseases [72,79,140]. Two examples of major global wheat virus epidemics are described below. 

#### 3.2.1. Yellow Dwarf Disease 

Yellow dwarf disease (YDD) causes the virus disease epidemic of greatest global significance for wheat (Table 1). It was reported first in 1951 in California, USA [141], and is the most widely distributed virus disease of wheat. It seriously damages wheat crops growing in all continents apart from Antarctica. It causes destructive epidemics in wheat crops growing in regions with temperate climates, as winter crops in regions with Mediterranean climates, and under the cooler conditions at higher altitudes in tropical and subtropical regions [4,72,75,76,142,143]. In wheat plants, YDD symptoms consist of leaf yellowing or reddening often most visible on the flag leaf, stiff leaves with an upright posture, diminished root growth, and plant stunting (Figure 1C,D). Heading is delayed, fewer grains form and these are shriveled [72,76,77,78]. Wheat grain yields are diminished by up to 60% and seed quality is greatly impaired [143,144,145]. Similar damaging YDD epidemics to those occurring in wheat also develop in barley and oat (*Avena sativa*) crops. In addition, maize, triticale (*Triticale hexaploide*) and Asian rice (*Oryza sativa*) crops are sometimes affected [4,75,76,77,78]. In their review of plant diseases threatening global food security, Strange and Scott [142] chose YDD as one of their examples of devastating plant virus diseases, emphasizing its worldwide distribution and debilitating effects on grain production. 

In wheat, YDD is caused by two different viruses, barley yellow dwarf virus (BYDV; genus, *Luteovirus,* family, *Luteoviridae*), and cereal yellow dwarf virus (CYDV; genus, *Polerovirus,* family, *Luteoviridae*). Their infections occur singly or in mixed infection. Both viruses cause the same types of YDD symptoms in infected wheat plants, and both are persistently aphid transmitted. Their most important aphid vectors are *Rhopalosiphum padi* (bird cherry-oat aphid), *R. maidis* (corn leaf aphid), *Sitobion avenae* (grain aphid) and *Schizaphis graminium* (wheat aphid). CYDV is transmitted by *R. padi*, whereas the main vectors of BYDV strains PAV, MAV, RMV and SGV are *R. padi* (PAV), *R. maidis* (RMV), *Sitobion avenae* (MAV) and *Schizaphis graminium* (SGV) [4,72,75,76,77,78,79,142]. Their respective aphid vectors spread them to wheat crops from alternative host infection reservoirs consisting mainly of infected wild, weed or pasture grasses, or volunteer cereals [4,72,75,76,77,78,79]. Viral ancestors of BYDV and CYDV likely emerged from infected wild grasses to infect wheat. Whether this occurred first in wheat’s center of domestication in the fertile crescent region, or has occurred one or several more times in different world regions, has yet to be revealed by phylogeographic or other evolutionary studies. However, recombination occurred frequently within BYDV, and therefore seems likely to have played a significant role in its adaptation to wheat and other cereal crop hosts and generating virulent new variants [146]. How BYDV and CYDV might have spread from one continent to another is unknown but one possibility is that they were introduced by viruliferous aphid vectors carried over long distances in wind currents [75].

The development of severe YDD epidemics varies seasonally. It depends upon weather conditions (rainfall and temperature) that promote early build-up of aphid vectors in alternative aphid and BYDV/CYDV hosts (grasses and volunteer cereals), and their large-scale migration to wheat crops at an early growth stage. When this occurs, widespread YDD develops causing significant losses. Mean daily rainfall and temperature data before sowing allow predictions of infection incidence and losses, and provide decision support over insecticide applications to kill aphid vectors [4,143,147]. Recently, epidemics of BYDV and CYDV in wheat crops have been controlled well by application of insecticides as seed dressings followed by within-crop foliar applications to kill their aphid vectors [144,148]. Widespread adoption of these control measures, especially in developed countries, has meant that, overall, epidemics caused by these two viruses are less damaging than in the past. However, in the future, this is likely to change with a return to the more destructive epidemics of the past [4,77,78]. This is because of (i) an increase in the numbers of their aphid vectors arising from withdrawal of neonicotinoid insecticides for use as seed dressings, and (ii) greater difficulties in timing foliar insecticide applications effectively due to the increasingly unpredictable global climate resulting from global warming [27,28,149]. 

#### 3.2.2. Wheat Streak Mosaic Disease

Wheat streak mosaic disease (WSMD) causes the second most important virus disease epidemic of wheat globally (Table 1). It was first reported in 1922 in the USA [83]. It infects wheat in most of the world’s main wheat-growing regions, including Australasia, Europe, the Middle East, Central Asia (Iran, Kazakhstan), East Asia (China), SSA (Nigeria, Zambia, South Africa), South America (Brazil, Argentina) and North America (USA, Mexico, Canada). It causes sporadic but disastrous epidemics in wheat in different world regions, and is the most destructive virus disease of wheat overall in the Great Plains region of North America, an area spanning 1.3 million km^2^. However, unlike YDD, which is most damaging when wheat crops are gown under cool conditions, WSMD is at its most destructive when they are grown under warm conditions [72,79,80,81,82,83,150]. Its foliage symptoms in wheat plants consist of yellow or pale green leaf streaking, tip yellowing of older leaves, a tufted growth habit and plant stunting (Figure 1E,F) [72,79,83]. It causes yield losses that reach 80–100% when infection is widespread early in the life of the crop. Moreover, like YDD, it also causes shriveled grain such that poor seed quality may render all wheat grain that remains unmarketable [72,79,80,81,82,83,150]. 

WSMD is caused by infection with WSMV, which was recently described as “a century old virus with rising importance worldwide” [83]. WSMV is eriophyid mite-transmitted, and its vector is the tiny wheat curl mite (WCM; *Aceria tosichella*). When viruliferous WCM are blown away from infected plants onto healthy plants by the wind, WSMV is transmitted to them [4,72,79,83]. The virus is seed-borne at a low level in wheat, and infected wheat seed not only plays a critical role in its survival between growing seasons but also in enabling its distribution around the world [81,82,151,152]. WSMV also infects barley, maize, oats, rye and sorghum, and some mostly annual grasses, but is not seed-borne in them [82,83]. In the Great Plains region of North America, annual autumn sowings made soon after previous wheat crops are harvested allow WCM to transmit WSMV from remaining infected volunteer cereal plants to emerging young wheat seedlings [72,79,80,83]. However, in regions with Mediterranean climates, in which wheat’s main growing period is winter and the summers are hot and dry, WSMV survives the dry conditions in spilt infected wheat seed or harvested wheat seed stocks. Both volunteer and sown wheat plants that germinate from these infected seeds act as primary WSMV infection foci for WCM to acquire the virus from and then spread it within the crop [81,82]. Epidemics of WSMV in wheat crops are favored by extended wheat growing periods (e.g., extended for graze-grain cropping) and growing wheat under warm conditions that favor build-up of its WCM vector. WSMV is currently controlled by combining (i) herbicide application to kill grasses and volunteer cereals 4 weeks before sowing with (ii) altering the sowing date to avoid the warmer conditions that favor WCM build-up, and (iii) sowing WSMV-free seed stocks [82,83,153]. The more erratic climate caused by global warming will likely render alterations to sowing dates less effective. Furthermore, although widespread adoption of such control measures is likely in developed countries, it seems less likely to occur, or be effective, in smallholder farms in developing countries where warmer temperatures favor increased WSMV spread by its WCM vector. 

An ancestor of WSMV most likely spread from infected wild wheat or other grasses to infect wheat when this crop was first domesticated. Due to the high temperature requirement (24–27 °C) for optimum activity of its WCM vector [27] and the likelihood of such temperatures occurring in wheat’s fertile crescent domestication center in the Middle East [138], its initial origin seems likely to be within this region. However, it might also have emerged one or several more times in different world regions. The answer to this question requires further phylogeographic or other evolutionary studies like those reviewed by Singh et al. [83], but including more geographically diverse WSMV isolates, particularly ones from the fertile crescent region. WSMV from Turkey is reported to have reached the USA in the late 1800s and then spread to the Great Plains region of the USA and Canada, and to Mexico [72,154]. Its recent introduction to Argentina in South America and Australia resulted from inadvertent distribution of WSMV-infected wheat seed by the international seed trade or as introductions of germplasm [72,152]. Its earlier spread to other world regions likely occurred similarly. Thus, as with MCMV in maize seed and MLND (see Section 3.1. above), the major global epidemic of WSMD in wheat provides an example of food security being threatened by the inadvertent introduction of a seed-borne virus to new world regions. 

### 3.3. Rice 

As a staple food crop, Asian rice (*Oryza sativa*) is ranked first and third in importance in developing countries and the world, respectively [66,155]. It is therefore of critical significance regarding future food security. Its center of crop domestication is China, where this process started approximately 10,000 years ago. It was distributed from China to the rest of East Asia, Southeast Asia, and the Indian subcontinent. After that, it was introduced to the Middle East, Europe and Africa, and within the more recent past to the Americas and Oceania [156]. The rice crop suffers from many virus diseases [84], e.g., the devastating rice virus disease pandemic described below. 

#### Rice Tungro Disease

Rice tungro disease (RTD) constitutes the most devastating virus disease of rice in tropical regions of Southeast Asia, southern China and the Indian subcontinent, where it causes a devastating pandemic [4,13,14,38,84,85]. Its name ‘tungro’ actually means ‘degenerated growth’ in Filipino. RTD is indigenous to tropical Southern Asia, where it has been known for many years, e.g., since 1869 in Indonesia, albeit named differently then. However, it was considered unimportant up until 1963 when devastating epidemics coincided with the ‘green revolution’ in tropical rice agriculture. Their occurrence was due to wide-scale adoption of high-yielding but highly vulnerable new rice cultivars bred by the International Rice Research Institute (IRRI), and the agricultural intensification associated with their use which enabled rice crops to be grown as dense stands in overlapping or close sequences [4,13,14,85]. These vulnerable new cultivars soon displaced the traditional cultivars grown before them. The disastrous RTD epidemics that followed resulted in famines in rice-growing regions across southern Asia, altering some countries into becoming rice importers rather than net rice exporters. The estimated loss in annual rice production reached US $1.5 billion annually [4,13,14,85]. In rice plants, ‘tungro’ disease symptoms include leaf yellowing or orange-yellowing, striping and mottling, diminished tillering and plant stunting, associated with production of partly filled or sterile grains. Yield losses in susceptible rice cultivars infected at an early growth stage reach 70–90% [157,158]. 

RTD is caused by mixed infection with RTBV and RTSV. Although plants infected with RTD develop severe foliage symptoms, plants infected solely with RTSV only show mild stunting, whereas those infected with RTBV develop mild ‘tungro’ symptoms. RTSV and RTBV are transmitted semi-persistently by several leafhopper vector species, the most efficient of which is *Nephotettix virescens* [4,84,85]. Infected plants of wild rice and grass weeds, which are often associated with rice paddies, are alternative hosts of both viruses. They, volunteer cultivated rice plants and surviving rice stubbles act as the principal reservoirs for RTSV and RTBV spread to rice crops. Most crop infection with these two viruses takes place after rice seedlings are transplanted, rather than before they are transplanted from nurseries [84,157]. However, spread of both viruses over long distances likely resulted from international trade in infected rice seedlings. 

The viral ancestors of RTBV and RTSV most likely emerged from infected wild rice or grasses to infect rice soon after this crop was domesticated. Phylogenetic analysis of RTBV isolates from India and Southeast Asian countries revealed two major phylogroups, one containing South Asian (=Indian sub-continent) and the other Southeast Asian isolates [61,62,84]. Similarly, phylogenetic analysis of a smaller number of RTSV isolates revealed a similar two phylogroup situation with separate South Asian and the Southeast/East Asian phylogroups [86]. Therefore, both RTBV and RTSV likely coevolved with rice within each of these regions, leading to the formation of separate South Asian and Southeast Asian (RTBV) or Southeast Asian/East Asian (RTSV) phylogroups. Moreover, recombination apparently played an important role in the evolution of both viruses [87,88]. As mentioned above, the introduction of high-yielding but highly vulnerable rice cultivars, and the agricultural intensification associated with their use, were crucial contributors to RTD pandemic development. In light of the global significance of rice as the developing world’s most important staple food crop, the RTD pandemic constitutes a critical threat to developing country food security. 

## 4. Root and Tuber Crops 

### 4.1. Potato

Approximately 9000 years ago, the potato (*Solanum tuberosum*) crop was domesticated from wild potato species native to the Lake Titicaca sector of the Altiplano in the Andean region of South America [159]. Potato land races were first brought from South America to Europe in the second half of the 16th century as part of the Colombian Exchange following the Spanish arrival in the Americas in 1542 [160], after which the crop was taken to other continents [161]. Potato now ranks as the world’s fourth most important staple food crop after maize, wheat and rice, and the third most important in food-insecure developing countries. Although generally more suited to cool conditions, over the last two decades its production increased considerably in developing countries, especially at higher altitudes. It now plays an increasingly important role in helping provide food security in the developing world [66,162,163]. Potato crops suffer from many virus diseases [89,90,164]. An example of a recent major global virus disease epidemic of potato is described below.

#### Potato Tuber Necrotic Ringspot Disease

**Figure 2:**
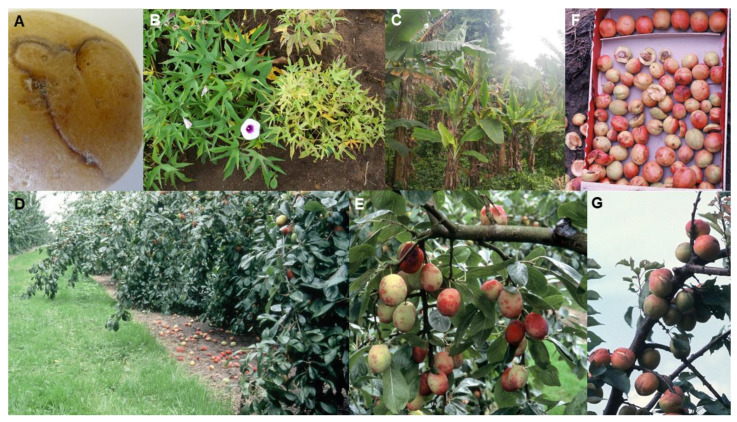
(**A**) Tuber of potato cultivar Nadine showing potato tuber necrotic ringspot disease (sunken necrotic rings and lines) typical of infection with R2 recombinant necrogenic strains of potato virus Y. (**B**) Sweet potato plant with sweet potato disease showing severe plant stunting and leaf chlorosis symptoms on right, healthy plant on left (image credit @International Potato Center/Segundo Fuentes). (**C**) Banana plantation with banana bunchy top diseased plants showing severe stunting, and short narrow upright leaves bunched at their tops, tall healthy plants on left side (image credit @International Institute of Tropical Agriculture/Lava Kumar). (**D**). Plum orchard showing premature fruit drop caused by plum pox disease. (**E**) Diseased fruit on plum tree showing ‘pock marks’ caused by plum pox disease. (**F**) Diseased apricot fruit harvested from apricot orchard showing ‘pock marks’ and internal flesh browning caused by plum pox disease. (**G**) Diseased apricot fruits splitting before harvest on plum pox diseased tree.

The following two paragraphs provide a brief historical account of PVY’s role in causing potato disease covering the period starting when the virus was first found up until its recent re-emergence as the cause of the current major global potato disease epidemic caused by its necrogenic R2 variants. These variants, which cause potato tuber necrotic ringspot disease (PTNRD; Figure 2A), arose by recombination between two of its strains [40,58,89,90,91,92,93].

After the potato was introduced to Europe in the second half of the 16th century, propagation of its tubers by replanting them continually year after year led to a devastating foliage degeneration disorder that diminished its tuber yields drastically. Until potato breeding from crosses between selected parental lines commenced in the 19th century [161], addressing this disorder necessitated frequent cultivar renewal involving growing seedlings from open-pollinated potato true seed, and selecting new cultivars from them. It was not until the early 20th century that the cause of the degeneration disorder was determined. It was found to result from mixed infection with PVY and one or more of several different potato viruses [165]. The most widespread and damaging virus complex consisted of mixed infection with PVY and potato leaf-roll virus (PLRV; genus, *Polerovirus*, family, *Luteoviridae*). However, PVY also formed complexes when present in mixed infection with two other common viruses, PVA and potato virus X (PVX; genus, *Potexvirus,* family, *Alphaflexiviridae*), the resulting synergistic interactions resulting in the severe foliage disorders ‘crinkle and ‘rugose mosaic’, respectively [89,90,164,166]. When present alone, PVY causes foliage symptoms that vary widely in severity depending upon which PVY strain and potato cultivar are involved, whether strain-specific hypersensitivity genes are present, and whether infection is current season derived or comes from planting infected tubers. Its symptoms range from severe shoot necrosis and plant death to severe leaf mottle/mosaic and deformation, mild leaf mosaic/mottle or entirely asymptomatic infection. Tuber necrosis that renders tubers unmarketable also develops as part of a strain-specific hypersensitive response in some combinations of virus strain and potato cultivar. Except when no tubers whatsoever form due to infected plants being killed by this type of response, infection with PVY alone decreases tuber yields by up to 80% [89,90,164,166,167]. PVY infection also causes serious diseases in several other solanaceous crop species, including tobacco, tomato, and pepper [8,168]. 

PVY is transmitted from plant to plant non-persistently by several aphid species, e.g., *Myzus persicae* (green peach aphid), by planting PVY-infected potato seed tubers, and, occasionally, by plant-to-plant contact [89,90,164,168,169]. Its alternative hosts include pepper, tomato, tobacco and many wild *Solanaceae* species [40,168]. It emerged in the Andean center of domestication of potato, and, after 1542, potato tubers infected with it were taken from there first to Europe via the Colombian Exchange, and from Europe to other continents [58,92,160,170,171]. In Europe, the advent of healthy seed potato stock schemes >80 years ago [166] combined with breeding new potato cultivars from parental plants with strain-specific PVY resistance genes *Ny* and *Nc*, elicited by its main biological strain groups PVY^O^ and PVY^C^, respectively [167,172], greatly limited its spread. However, in the 1930s an additional strain, PVY^N^, spread from South America to Europe. It caused obvious veinal necrosis symptoms in tobacco plants, but its subtle symptoms in potato foliage often went unnoticed, such that infected plants were missed during seed potato field inspections. It also overcame hypersensitive resistance genes *Ny, Nc* and additional PVY resistance gene *Nz*. These characteristics resulted in its widespread distribution through large-scale inadvertent planting of PVY^N^-infected seed potato stocks [89,92,166,167]. Coinfection of potato plants with PVY^O^ and PVY^N^ eventually led to recombination between these two stain groups [40,89,91]. This resulted in formation two recombinant PVY phylogroups, R1 and R2, both of which overcame resistance genes *Ny* and *Nc,* [92], but R2 failed to overcome gene *Nz* [40]. These R1 and R2 populations also caused subtle foliage symptoms in potato plants, so, as with PVY^N^, they too were often missed in seed potato field inspections. In addition, their aphid transmission was more efficient. These properties favored their inadvertent widespread dissemination on a global scale within infected seed potato stocks [40,89,91]. Moreover, the R2 population also caused PTNRD, a tuber quality disorder that had very serious repercussions due to the lack of marketability of affected potato tubers (Figure 2A) [40,89,91,92]. 

PVY’s R2 phylogroup was found first in the early 1980s in Europe, but its R1 phylogroup apparently arose earlier [92]. Apart from within the crop’s Andean domestication center, PVY phylogroups R1 and R2, but especially R2, have now largely displaced non-recombinant biological PVY strain groups PVY^O^, PVY^C^ and PVY^N^ in most potato-growing regions of the world [40,58,92,172,173,174]. The widespread global occurrence of PTNRD caused by R2 and the current inability to manage it adequately through healthy seed potato stock schemes, have resulted in a global PTNRD epidemic in potato crops grown for human and livestock consumption [40,89,90]. Therefore, this major epidemic caused by necrogenic PVY provides an example of global food security being threatened by virus disease re-emergence arising from generation of virulent new virus variants with altered pathogenicity. 

### 4.2. Sweet Potato

Sweet potato (*Ipomoea batatas*) was domesticated approximately 10,000 years ago. Its domestication occurred twice, once each in Central and South America, the two gene pools subsequently becoming mixed. In the pre-Colombian era, it was taken from the Americas and dispersed widely around Polynesia and from there to Melanesia [175,176]. In the post-Colombian era, it was distributed to Southeast and East Asia, and to Europe, in the 1500s, and subsequently to other regions of the world with warm climates, including SSA [177]. The crop is now widely grown in all regions of the world with warm climates, including in Africa, Asia, Oceania and the Americas, the largest production being concentrated in China and East African countries. Amongst staple food crops, sweet potato is ranked seventh in importance globally and fifth most important for developing countries [66,163,178,179]. It constitutes an ideal crop for many subsistence agriculture situations as it tolerates infertile soils and drought well, and its tuberous roots can be left stored in the soil for long periods before harvest [94,95]. Sweet potato crops become infected with many viruses [95]. An example of a devastating global sweet potato virus disease pandemic is described below.

#### Sweet Potato Virus Disease 

Sweet potato virus disease (SPVD) causes the most devastating virus disease of the sweet potato crop. It is caused by mixed infection with the sweet potato chlorotic stunt virus (SPCSV; genus, *Crinivirus*, family, *Closteroviridae*) and a member of the *Potyviridae* family. The latter virus is usually sweet potato feathery mottle virus (SPFMV; genus, *Potyvirus* *family, Potyviridae*) or sweet potato mild mottle virus (SPMMV; genus, *Ipomovirus,* family, *Potyviridae*). A synergistic interaction between SPCSV and SPFMV or SPMMV causes a very damaging disease consisting of severe leaf mosaic and malformation and plant stunting, and a tuberous root yield reduction of >85% (Figure 2B) [94,96]. However, on their own, these three viruses cause milder foliar symptoms and have less impact on tuberous root yields, especially SPFMV and SPMMV [94]. Globally, sweet potato crops are frequently SPFMV-infected, SPCSV is the next commonest virus and SPMMV the third commonest. SPCSV consists of two strains, East African (EA) and West African (WA). SPCSV-EA occurs in East Africa, Madagascar and parts of South America. SPCSV-WA is found in West Africa and most other sweet potato-growing regions of the world. SPMMV occurs in East and South Africa, Southeast Asia, New Zealand and Egypt [94,97,99,180]. SPVD is widely distributed in warmer sweet potato-growing regions of the world, occurring wherever SPCSV is found. Its distribution includes SSA, North Africa, Middle East, Southeast Asia, East Asia, and North, Central and South America [38,94,98,100,181,182,183,184]. However, currently the SPVD pandemic in East Africa, where 75% of the crop’s African sweet potato production occurs, is causing the greatest concern. The countries suffering devastating losses include Uganda, Kenya, Rwanda and Tanzania. In the past, in the Democratic Republic of Congo, Central Africa, the losses from SPVD were so severe that they stopped growing sweet potato altogether [94,97]. 

SPCSV and SPMMV are transmitted semi-persistently by the whiteflies, SPCSV by *Bemisia tabaci* (silver leaf whitefly) and *Trialeurodes abutilonea* (glasshouse whitefly) and SPMMV by *B. tabaci*. SPFMV is transmitted non-persistently by the aphid species *M. persicae* and *Aphis gossypii* (cotton aphid) [72,99]. SPFMV likely coevolved with cultivated sweet potato in one of the crop’s two domestication centers and was distributed globally from the Americas in infected tuberous roots of sweet potato. However, East Africa is considered a secondary ‘hot spot’ for its diversification due to widespread infection of alternative host species and recombination between SPFMV isolates occurring locally [185]. The same applies to SPCSV too as, although it most likely also originated in cultivated sweet potato in one of the crop’s two domestication centers, widespread infection of alternative host species suggest that, later on, East Africa was a secondary ‘hot spot’ for its diversification [186,187]. By contrast, SPMMV is considered to have emerged after cultivated sweet potato’s introduction to East Africa. This was by ancestral virus spillover from alternative host species in the *Convolvulaceae* that it infects naturally in this region [188]. Alternative hosts of SPCSV, SPFMV and SPMMV include the wild *Convolvulaceae* species *Ipomoea* spp. (several species), *Hewittia sublobata*, and *Lepistemon owariensis* [185,188,189]. 

What was responsible for the emergence of SPVD as a major threat to sweet potato production within East Africa? The traditional East African land races that survived since sweet potato first arrived were tolerant of SPVD. What led to the SPVD pandemic in that part of the world was (i) land race displacement by vulnerable high-yielding sweet potato cultivars bred elsewhere and (ii) agricultural intensification aimed at growing more sweet potato to help meet the needs of the burgeoning human population [96,187]. Thus, widespread adoption of vulnerable high-yielding sweet potato cultivars resulted in devastating consequences for food-insecure smallholder farmers many of whom had little choice but to give up growing sweet potato or grow lower-yielding but safer traditional cultivars. 

## 5. Plantation and Orchard Crops

### 5.1. Banana 

As a world staple food crop, banana (*Musa* spp., including dessert banana and plantain) ranks sixth in the world after maize, wheat, rice, potato and cassava, and the fourth most important for developing countries [66,163]. It is grown widely in the humid tropical and subtropical regions of the Americas, Africa, Asia and Oceania. It is a perennial herb that grows up to 3 meters high, but lacks the lignification and secondary stem thickening typical of trees. It was first domesticated 10000 to 7000 years ago. Although its main center of diversity is considered to be Malaysia and Indonesia, its center of domestication spans a region ranging from the Indian subcontinent eastwards to Polynesia. Human trade and migration have played a major role in its wide-scale dissemination elsewhere in the world [190,191]. Although most widely grown cultivars have seedless fruits, banana arises from crosses involving two wild species that produce seeds, *M. acuminata* (genetic constitution AA) and *M. balbisiana* (BB) [191]. An example of a devastating banana virus disease pandemic of worldwide importance is described below.

#### Banana Bunchy Top Disease

Banana bunchy top disease (BBTD) causes a devastating pandemic in banana crops. It constitutes the most damaging virus disease affecting this crop on a global scale and seriously threatens food security in developing countries [63,64,192]. BBTD was first recorded in 1889 in Fiji, Oceania. The crop is propagated vegetatively and the disease has been disseminated widely through human activities that unknowingly transported infected planting material between countries, and from one continent to another. Its current distribution includes all tropical and sub-tropical regions of the world: SSA and North Africa, Middle East (Iran), Indian subcontinent, Southeast Asia, East Asia, Oceania (including Australia). It reached SSA in the 1950s, where it subsequently became very widespread and destructive, but is still absent in banana producing regions of the Americas [38,63,64,72,101,192]. BBTD-resistant cultivars are lacking, but some cultivars are tolerant of infection. BBTD epidemics in widely grown vulnerable cultivars always result in major reductions in banana fruit production and areas planted. In regions with widespread infection, their cultivation is often abandoned. Once established, BBTD is very difficult to remove from affected plantations and regions. Drastic measures are required to achieve this, including widespread destruction of infected plantations and completely preventing the distribution of banana planting material within and outside of BBTD-affected districts [63,64,192]. Fruits are rarely produced by BBTD-affected plants, but the few that occasionally develop are small and deformed, 100% yield losses often occurring. The most obvious foliage symptom from which the disease gets its name is the clusters of short, narrow, brittle upright leaves bunched at the top of the plant (Figure 2C). Other foliage symptoms include dark green flecking and streaking of leaves, and severe stunting of suckers [63,64,192]. 

BBTD is caused by BBTV, which is transmitted persistently by *Pentalonia nigronervosa* (banana aphid). This aphid vector spreads BBTV locally within and between banana and plantations. Its natural alternative hosts are limited to three other *Musaceae* species, *Musa paradisiaca, M. textilis* and *Ensete ventriculosum*. These three species are potential sources for BBTV to spread locally to plantations [64]. Phylogenetic analysis of BBTV’s DNA-R component sequences revealed two major phylogroups: the Pacific-Indian Oceans group (PIO) consisting of isolates from Africa, Oceania, Myanmar and the Indian subcontinent, and the Southeast Asian group (SEA) consisting of isolates from East and Southeast Asia. BBTV’s likely origin is by spread of an ancestral virus from its alternative hosts within the crop’s original center of domestication where BBTV later split into the geographically based PIO and SEA. Its occurrence elsewhere occurred due to introduction of infected planting material carrying the PIO phylogroup to Africa and the SEA phylogroup to East Asia. BBTV’s absence in the Americas can be explained by introductions having consisted solely of healthy planting material [64]. What triggered the emergence of BBTV as the cause of an extremely destructive pandemic in tropical regions of many developing countries, especially those in SSA? The most likely reasons are wide-scale inadvertent transportation of BBTV-infected planting material of vulnerable bred banana cultivars to new geographic locations, frequent new introductions of its *Pentalonia nigronervosa* vector and agricultural intensification to increase production. 

### 5.2. Citrus Fruit

Citrus fruits are important for human nutrition. They provide the human body with vitamins, minerals and plant compounds that bestow health benefits, including anti-inflammatory and antioxidant effects. They include orange, mandarin, lemon, grapefruit, lime and pomelo. The center of origin of wild *Citrus* species is considered to be in the southeast foothills of the Himalayas from which they spread throughout subtropical and tropical regions of the Indian subcontinent, East Asia, Southeast Asia, Melanesia, and Oceania [193]. The different citrus crops were most likely domesticated at least 5000 years ago from wild *Citrus* species in different parts of this wider region, especially in Southeast Asia, before being introduced gradually to all continents except Antarctica [194]. Citrus crops suffer from a range of virus and virus-like diseases [195]. The example below describes a devastating citrus virus disease pandemic. 

#### Citrus Tristeza Disease

Citrus tristeza disease (CTD) is the most economically important virus disease of citrus plantations globally. It causes a lethal tree decline syndrome, especially in oranges, mandarins, grapefruits and limes propagated on sour orange rootstocks. At the beginning of the 20th century, death of trees growing on sour orange rootstocks was reported in South Africa, followed by Argentina and Brazil in the 1930s, and shortly afterwards in several other South American countries. Its name ‘tristeza’, which means ‘sadness’ in Spanish and Portuguese, was given it due to the massive destruction it engendered in South American plantations. CTD was first recorded in the 1930s in Argentina and shortly afterwards in Brazil and several other South American countries. Virtually all citrus trees growing on sour orange rootstocks were killed and plantations abandoned in worst-affected regions. By 1959 in Brazil’s Sao Paulo state, 75% of all orange trees (i.e., 6 million trees) were killed, and similar problems occurred in South Africa, West Africa and California [4]. After 1980, infected citrus plants from countries where CTD was endemic were inadvertently shipped in vast numbers to CTD-free countries, and a large-scale introduction effort that exchanged citrus germplasm between countries further expanded the pandemic. CTD now occurs in South, Central and North America, SSA and North Africa, Europe, Middle East and Arabia, Indian subcontinent, East Asia, Southeast Asia and Oceania [72]. Worldwide, CTD has killed more than 100 million trees in Argentina (>26 million), Brazil (>6 million), Venezuela (>6 million), the USA (>3 million), Spain (>40 million), South Africa since 1910 and Israel since the 1950s, and in many other countries with warmer climates across the globe (many millions more killed). Collectively, CTD epidemics have caused a devastating global pandemic in orange, mandarin, grapefruit and lime orchards and destroyed entire industries. Different phases of this pandemic have been described in many review articles and research papers [4,101,102,103,104,105,106,107,108,109,110,195,196]. 

CTD results from killing of the phloem below the graft union causing foliage symptoms consisting of leaf size reduction and veinal yellowing, leaf drop, twig dieback, wilting and tree death. This tree decline followed by death occurs at different rates. There is no fruit yield with ‘quick decline’, and although some small fruit may form with ‘slow decline’, it still decreases fruit yields drastically. In addition, the related stem pitting decline syndrome (CSPS) severely damages plantations of grapefruit, lime and several orange cultivars, and another related condition, seedling yellows syndrome (CYS), also damages sour orange, lemon and grapefruit plants mostly in nurseries. With CSPS, citrus branches and trunks develop pits and grooves, and gum often appears, and the affected trees are stunted producing fewer, smaller fruit. Thus, tree vigor and fruit yield are both diminished. With CYS, yellowing develops on leaves and branches die back [72,102]. 

The causal agent of CTD, CSPS and CYS in citrus trees is citrus tristeza virus (CTV; genus, *Closterovirus*, family, *Closteroviridae*). A range of biological CTV strains exist that elicit symptoms which vary in severity from quick tree decline and death to asymptomatic infection, and in type (tree decline, CSPS, CYS). Symptom severity also differs with citrus cultivar and rootstock type, and is favored by warmer conditions. CTV is transmitted semi-persistently by several aphid species. Its most efficient and globally important vector is *Toxoptera citricida* (brown citrus aphid). However, although less efficient, *A. gossypii* is the most important vector in Europe, and two other less efficient vectors are also of importance in other continents, *T. aurantii* (black citrus aphid) and *A. spiraecola* (green citrus aphid). Viruliferous aphids spread the virus locally within and between citrus plantations, and, over greater distances when strong winds disperse them. However, more distant CTV spread, including between regions, countries and continents, is almost entirely by distribution of infected planting material (rootstocks, grafted trees and scions) derived from CTV-infected nurseries. Alternative natural CTV hosts are limited to other citrus species and species in related genera, such as *Fortunella* and *Poncirus* [4,72,102,103,104,105,106,107,108,109,110,196]. 

CTV, its *T. citricida* vector and citrus fruit crops coevolved together within Southeast Asia, and probably also within its wider domestication center in the Indian subcontinent, East Asia, Melanesia, and Oceania. Before 1890, long-distance dispersal of citrus propagules only involved fruits and seeds. Although CTV and *T. citricida* both spread to nearby countries with common land borders, no long-distance dispersal occurred during that period. After 1890, advancements in maritime shipping transport arrangements enabled trade in citrus plants growing in terrariums to commence. This enabled both CTV and *T. citricida* to disperse to distant continents. For example, as CTD had appeared in South Africa by 1910, CTV and *T. citricida* must have arrived before then. They both reached Argentina and Brazil in South America by the 1930s, albeit inadvertently, through citrus plant trade or citrus germplasm introductions. Over a long period before then, in these two countries very extensive orange plantations had existed without suffering from CTD despite the practice of growing trees grafted onto sour orange rootstocks. Moreover, before CTV and *T. citricida* arrived, a similar situation occurred in many other subtropical or tropical world regions. However, soon after CTV and *T. citricida* arrived, a massive global CTD pandemic developed [4,72,102,106,107,196]. 

In addition to the distribution of huge amounts of citrus planting material some of which was CTV infected and *T. citricida* infested, another factor that contributed to CTD’s appearance far away from the citrus domestication center was the widespread growing of citrus trees derived from CTV-vulnerable cultivar scions grafted onto CTV-susceptible sour orange rootstocks. In recent times, because CTV-tolerant rootstocks prevent phloem death below the graft union, their widespread adoption as a replacement for CTV-sensitive sour orange rootstocks has helped diminish CTD’s impact. However, where healthy citrus stock schemes are lacking, CTV-tolerant rootstocks still fail to prevent CSPS from occurring. Therefore, CSPS remains an important limitation to optimizing citrus plantation productivity. Regions without healthy citrus stock schemes mostly involve countries that are food insecure so such countries need to develop such schemes to overcome this syndrome [4,72,102,106,107]. 

### 5.3. Stone Fruit

Like citrus fruits, stone fruits (*Prunus* spp.) are also important for human nutrition, not only as a source of vitamins and minerals but also generally as part of healthy eating patterns that reduce the risk developing certain chronic diseases that afflict humanity. They include the *Prunus* species plums, cherries, peaches, nectarines, apricots, cherries and almonds. Cultivated *Prunus* species were all domesticated in the northern hemisphere, for example plum and cherry species in Europe, Asia and North America, but peaches and nectarines in China [197]. They suffer from a wide range of virus diseases [198]. An example of a devastating stone fruit virus disease pandemic of global importance is described below. 

#### Plum Pox Disease 

Plum pox disease (PPD = Sharka disease) is the most destructive virus disease of stone fruit worldwide. Collectively, its epidemics have caused a devastating pandemic in plum, peach, apricot and nectarine orchards, which, over the last 40 years, has been the subject of numerous reviews [4,111,112,113,114,115,116]. PPD was reported first in 1915 in Bulgaria, southeast Europe. It subsequently spread widely in Europe. Until 1992, there were no reports of it from other continents. However, its spread elsewhere occurred soon afterwards. It is now present throughout Europe, and has been found in most countries in the Middle East, the Indian subcontinent and East Asia, and two countries each within North Africa (Egypt, Tunisia), South America (Argentina, Chile) and North America (Canada, USA); its eradication from the USA was reported recently but it is still present just across the northern border in Canada. PPD epidemics cause enormous losses due to premature fruit drop and unmarketable fruit. In diseased orchards and commercial nurseries, very large-sale destruction of trees has occurred in attempts to eradicate or contain the disease. Its symptoms vary from mild to obvious depending on cultivar sensitivity to infection, and diseased fruits often drop (Figure 2D). Plum and apricot fruits are deformed and their flesh develops internal browning visible as ‘pock marks’ on their surfaces hence the name ‘plum pox’ (Figure 2E,F). Apricot fruits sometimes split (Figure 2G). Peach and nectarine fruit develop pigmented surface line patterns or rings. Foliage symptoms are variable in intensity and generally most severe in plums. They consist of leaf vein clearing, chlorotic spots or rings, chlorosis and deformation. Infection of sensitive cultivars causes up to 80–100% losses in stone fruit yields. In addition to decreasing fruit yield and quality, infected trees may produce no saleable fruit and the overall productive lifespan of infected orchards is greatly diminished [72,111,112,113,114,115,116]. 

The causal agent of PPD is PPV, which is transmitted non-persistently by many aphid species several of which are important vectors, including *M. persicae*. These aphid vectors contribute to local PPV spread within and between orchards. More distant spread, including between regions, countries and continents, occurs mostly through the inadvertent distribution of infected planting material (rootstocks, grafted trees and scions) derived from PPV-infected nurseries. Strict quarantine regulations and sole use of healthy planting material are critical measures that prevent its long-distance spread. Apart from occurring in cultivated *Prunus* stone fruit and ornamental trees, natural infection with PPV also occurs in wild *Prunus* species [4,72,114,115,116]. Phylogenetic studies with PPV genomic sequences reveal that the center of diversity of PPV is central and eastern Europe and the Levant in the Middle East, which coincide with part of the plum and cherry crop domestication center [60,197]. Thus, PPV’s origin likely arose from spread of an ancestral virus infecting its wild *Prunus* hosts to plum and cherry within this region. By contrast, as peach and nectarine were domesticated in China, PPV’s spread into them required a new encounter situation when they were planted next to PPV-infected plum or cherry orchards following their introduction to Europe or the Levant. What caused PPV to emerge as the cause of such a destructive global pandemic? The most likely causes are wide-scale inadvertent transportation of PPV-infected *Prunus* planting material and germplasm to new geographic locations around the world, local spread by aphid vectors and the widespread adoption of vulnerable stone fruit cultivars [8,14]. 

## 6. Grain Legumes

Including grain legumes in the human diet is important for balanced nutrition and helping combat food insecurity. In addition, the atmospheric nitrogen they fix is important for soil fertility and achieving sustainable agriculture [199]. However, unreliability in obtaining adequate seed yields often experienced due to virus diseases is a critical factor that prevents their adoption on a broader scale. This applies not only to cool season grain legumes but also to grain legumes adapted to hot climates [117,118,200,201]. An example of a devastating major virus disease epidemic that afflicts the grain legume faba bean (*Vicia faba*) is described below.

### Faba Bean Necrotic Yellows Disease

Faba bean necrotic yellows disease (FBNYD) epidemics cause severe yield losses and crop failure in faba bean crops. FBNYD was reported first in Syria in 1986 [120], but now occurs in 17 different countries spanning a region extending eastwards from Spain in Europe to Pakistan in the India subcontinent. Its distribution not only includes these two countries at the opposite extremities of its range along with most countries in North Africa and the Middle East, but also includes Yemen in Arabia and countries extending southwards from Egypt to the horn of Africa [72,117,119,120]. FBNYD is the most economically important virus disease of faba bean in most of these countries [117]. For example, its epidemics were so devastating in Middle Egypt in the early 1990s that Egyptian faba bean production was forced to move northwards to the Nile Delta [117]. Foliage symptoms in faba bean plants start as small cupped young, and rolled older, leaves associated with severe plant stunting, but later the infected leaves develop interveinal chlorosis that becomes necrotic, followed by plant death. Seed yield losses are up to 90% [72,117]. Thus, its sporadic occurrence, widespread distribution over three continents and devastating impact on faba bean production, together warrant its designation as a major virus disease epidemic. 

FBNYD is caused by faba bean necrotic yellows virus (FBNYV; genus, *Nanovirus*, family, *Nanoviridae*), which is transmitted persistently by three aphid species, *A. craccivora, A. fabae* (black bean aphid) and *Acyrthosiphon pisum* (pea aphid). Although faba bean is FBNYV’s main host, its alternative hosts include the grain legume crops common bean (*Phaseolus vulgaris*), cowpea (*Vigna unguiculata*), chickpea (*Cicer arietinum*) and lentil (*Lens culinaris*), several wild and pasture legumes, and several non-legume species belonging to the genus *Amaranthus* [72,117]. FBNYV is an indigenous virus of the Middle East and North Africa region [117]. Moreover, the center of domestication of faba bean is the Middle East [202]. Therefore, both virus and crop likely coevolved together in the Middle East rather than FBNYV having resulted from a new encounter scenario that arose after introduction of faba bean outside its domestication center. The question arises as to why FBNYV emerged as a devastating pathogen of faba bean over the last three decades despite remaining unrecognized before then. A change to growing more vulnerable faba bean cultivars, especially in Middle Egypt [117], combined with agricultural intensification to boost production seem likely causes, but there may well be others. 

## 7. Annual Horticultural Crops 

This section describes three examples of devastating major virus disease epidemics caused by seed-borne viruses introduced by the international seed trade to new countries in the same continent or within other continents [7,16,19,30,31,203,204]. 

### 7.1. Tomato

Tomato (*Solanum lycopersicum*) is the most important vegetable crop worldwide [66]. Its nutritional value for the human body is because it supplies an important source of vitamins, minerals, antioxidants and other beneficial plant compounds. Shortly after humans first arrived in the central region (Ecuador and Peru) of the Andean region of South America, a semi-domesticated tomato variant derived from the wild tomato species *Solanum pimpinellifolium* was used as a food. It was then taken north within the Americas reaching Mexico approximately 7000 years ago, where the crop’s domestication continued. In the 16th century domesticated tomato was transferred from Mexico to Europe before being distributed worldwide [205,206]. The tomato crop becomes infected by >136 viruses, some of which cause very damaging diseases [127]. Two examples of recent major seed-borne tomato virus disease epidemics are described below. 

#### 7.1.1. Tomato Brown Rugose Fruit Disease 

**Figure 3:**
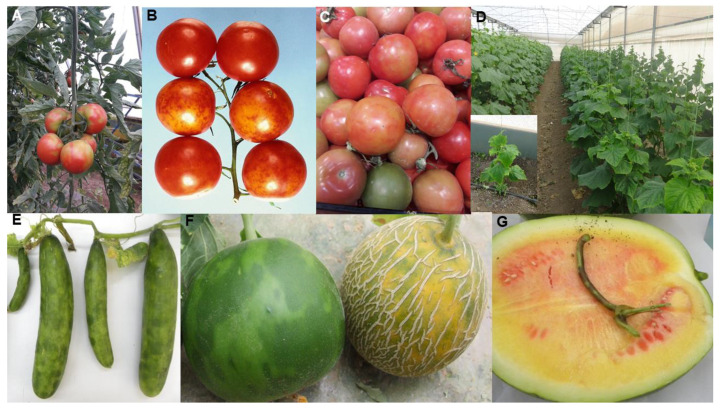
(**A**) Tomato brown rugose fruit diseased tomato fruit showing symptoms of uneven ripening with yellow and brown surface blotching (image credit @Volcani Center/Aviv Dombrovsky). (**B**) Pepino mosaic diseased tomato fruits infected with a severe pepino mosaic virus strain showing surface yellow marbling and discoloration (image credit @Dutch National Plant Protection Organization/Marlene Botermans). (**C**) Harvested tomato fruits showing severe yellow marbling symptoms that developed when initial infection with tomato brown rugose fruit mosaic virus was followed by later infection with a pepino mosaic virus mild strain commonly used commercially to provide cross protection against its severe strains (image credit @Volcani Center/Aviv Dombrovsky). (**D**) Cucumber plants growing in tunnel house: plants on right show symptoms of cucumber green mottle mosaic disease (chlorotic mosaic on leaves and plant stunting), plants on upper left are vigorous and healthy (insert shows early infected plant with severe stunting, reduction in leaf size and chlorotic mosaic). (**E**) Cucumber fruits with chlorotic mottle caused by cucumber green mottle mosaic disease (image credit @Volcani Center/Aviv Dombrovsky). (**F**) Fruits of honeydew melon with chlorotic mottle (left) and rockmelon with yellow mottle (right) caused by cucumber green mottle mosaic disease (image credit @Volcani Center/Aviv Dombrovsky). (**G**) Watermelon fruit with cucumber green mottle mosaic disease showing yellow spongy flesh and stem with necrotic lesions (image credit @Volcani Center/Aviv Dombrovsky. Images (**E**–**G**) all modified from [39].

Tomato brown rugose fruit virus (ToBRFV; genus, *Tobamovirus*, family, *Virgaviridae*) was first found in 2014 infecting tomato in Jordan so, apparently, it only emerged recently [207]. Since then, it has been found elsewhere in the Middle East (Israel, Turkey), and in European countries (Germany, Greece, Cyprus, Czech Republic, France, Italy, Poland, Spain, the Netherlands, UK), East Asia (China), North America (Mexico, USA), South America (Chile) and North Africa (Egypt, Sudan). In these countries, it is currently causing a serious virus disease epidemic, particularly in tomato crops grown under protected cropping [121,122,123,124]. It differs from the tomato infecting tobamoviruses tomato mosaic virus (ToMV) and tobacco mosaic virus (TMV) in being able to infect tomato plants carrying virus resistance gene *Tm-2^2^*, so all TMV- and ToMV-resistant tomato cultivars become ToBRFV infected [208]. A mutation or a recombination event likely occurred that broke tomato resistance gene *Tm-2^2^*, a gene that had apparently remained effective against tobamovises during the previous 55 years [209]. The main foliage symptoms of ToBRFV in tomato are leaf chlorosis, mosaic and mottling, and necrotic spotting on petioles and calyces. In tomato fruits, they are deformation, uneven ripening, yellow or brown spotting/botching and rugosity (Figure 3A). These symptoms render diseased tomato fruit unmarketable. ToBRFV was estimated to cause an overall 30%–70% loss in marketable yields of tomato fruit in Florida, accounting for an annual economic impact USD$262 million a year [124]. However, although underway, studies quantifying gross tomato fruit yield losses are yet to be completed. In addition, ToBRFV also infects pepper (*Capsicum annuum*) plants in which its foliage symptoms include leaf vein clearing, mosaic and discoloration, stem browning, and fruit mosaic and distortion [210]. However, it induces a hypersensitive resistance response in pepper cultivars carrying tobamovirus resistance genes *L1*, *L2* and *L3* [208] so this crop seems less threatened than tomato. 

ToBRFV has rod-shaped stable virions that reach high concentrations in infected plants and remain infectious for long periods on contaminated surfaces. These properties enable its efficient contact transmission when a healthy tomato plant comes into contact with an infected plant, contaminated soil, contaminated nutrient solutions or a contaminated surface, such as equipment, tools, hands and clothing used during pruning or trellising within protected cropping situations. It is also transmitted by grafting, cuttings [122,123,124] and bumblebee pollinators [211]. As with TMV and ToMV, it is seed-borne at low levels when tomato seeds become surface contaminated with its virions. Seed transmission results from contact between virion-contaminated seeds coats and young seedlings [123]. Trade in contaminated tomato seeds, combined with trade in infected seedlings and fruits, inadvertently results in its distribution within countries, to neighboring countries and, especially with contaminated seeds, over much greater distances internationally [124]. Potential alternative ToBRFV hosts identified by sap inoculation include eggplant (*Solanum melongena*), petunia (*Petunia hybrida*) and the weed *S. nigrum* [208]. Its epidemiology is incompletely understood but is being investigated currently [123].

What is responsible for ToBRFV’s apparent emergence in 2014 in the Middle East, followed by its rapid geographical dispersal to Europe, Africa, East Asia and the Americas over the last six years? One possibility is that it emerged recently from spread by contact transmission from an unknown infected alternative solanaceous crop or weed host to the introduced crop tomato, and was detected infecting tomato soon afterwards. Tomato seed crops then became ToBRFV-infected and the international seed trade distributed contaminated seed around the world, albeit inadvertently, introducing the virus to other counties and continents. Another possibility is that it was being confused with TMV or ToMV, and had already been present infecting tomatoes in all of these different parts of the world but had gone unnoticed [122]. However, tomato virus diseases have been studied intensively for many years, especially in Europe and North America [127]. Therefore, for this second scenario to be correct, its earlier worldwide presence in tomato cultivars would have to have remained undetected for a very long time. 

#### 7.1.2. Pepino Mosaic Disease

**Figure 4:**
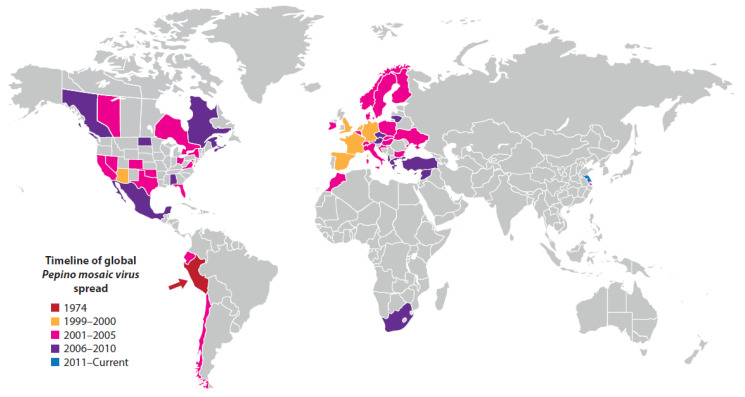
Global spread of pepino mosaic virus after its first detection in Peru, South America in 1974 (red arrow). After a 25 year delay, it reappeared in Western Europe, North America and East Asia. During the following decade, it spread rapidly within Europe and North America, arrived in some Middle Eastern and African countries, and was detected in two countries neighboring Peru. Subsequently, the only spread recorded was within China. The virus, which is seed-borne, was spread inadvertently by the international seed trade. An subsequent focus on trade in heathy tomato seed stocks reduced the risk of further global spread (figure credit @Washington State University/Naidu Rayapati). Figure from Supplementary data in [9].

Pepino mosaic virus (PepMV; genus *Potexvirus*, family, *Alphaflexiviridae*) was isolated first in 1974 from a plant of the indigenous Andean fruit crop pepino (*Solanum muricatum*) showing yellow leaf mosaic symptoms growing in coastal Peru. When inoculated with PepMV-infective sap, a range of other solanaceous crop species became infected, including tomato [125]. No further reports of PepMV occurred until 25 years after its initial isolation when it reappeared infecting tomato plants growing under protected cropping conditions in the Netherlands [212]. Thereafter, it soon spread to tomato crops in most European countries, North America (Canada, Mexico, USA), the Middle East (Israel, Syria, Turkey), Africa (Morocco, South Africa), and East Asia (China), and was also detected in two of Peru’s neighboring countries (Ecuador, Chile) (Figure 4) [72]. In addition to tomato foliage symptoms of leaf mosaic, chlorotic spotting, narrowing, distortion and reduced size, it caused fruit symptoms that reduced marketable yields, including surface marbling, bleaching and discoloration, splitting and uneven ripening (Figure 3B). However, although marketable yield losses can be up to 38% (depending upon PepMV strain, environmental conditions and the extent poor quality fruit can be sold), its effects on gross fruit yields are relatively minor, only reaching 5–10%. Nevertheless, since tomato constitutes >70% of the value of fresh vegetables produced worldwide and PepMV is not only highly contagious but also reaches very high infection incidences in tomato crops, by 2010 the marketable yield losses it caused in protected cropping had established it as one of the most threatening virus diseases of vegetables [127,128].

PepMV has stable rod-shaped virions and is transmitted readily by contact [125]. These properties enable it to be efficiently contact transmitted when a healthy tomato plant comes into contact with an infected plant, or a contaminated surface, such as tools, hands and clothing. This is particularly the case within protected cropping situations. Further, bumblebees spread the virus when used for pollination in tomato production, and it is readily seed-borne via tomato seed surface contamination. This seed contamination made possible its worldwide dissemination though distribution and sowing of contaminated commercial tomato seed stocks [7,126,127,128]. In addition to infecting pepper, PepMV has other natural alternative hosts including four wild tomato species in Peru, and 18 weed species belonging seven different plant families in Spain [213,214]. PepMV probably emerged initially in Peru or Ecuador by ancestral virus contact transmission from wild tomato species to land races of tomato, pepper and pepino undergoing domestication there. Although first found in Peru in 1974, PepMV is still considered an emerging rather than re-emerging virus outside South America. This is due to its sudden appearance infecting tomato in 1999 in Europe followed by its rapid international spread. Its arrival in other counties and continents was attributed to international seed companies using the central Andean region to multiply tomato seed crops. They began doing this because it enabled seed production to continue all year round, thus helping to satisfy the escalating global demand for tomato seed. The enhanced speed of international trade arising from transport by airplane assisted this process [7,127,128]. Tomato seed crops growing in the Andean region would most likely have acquired PepMV due to its spread by contact transmission from infected solanaceous crop or wild tomato hosts growing nearby, resulting in contamination of the seed harvested. Thus, given the global significance of the tomato crop, PepMV provides an example of the global food supply being threatened by major epidemic initiated by seed crop multiplication in South America. In addition, global trade in tomato fruits and seedlings, and possibly pepino cuttings, might also have contributed to PepMV’s worldwide distribution [126,127]. 

PepMV is managed in tomato crops by planting healthy tomato seeds and seedlings and applying strict hygiene precautions to prevent its spread [127]. Alternatively, since no PepMV-resistant tomato cultivars are available commercially, cross protection involving inoculating young tomato plants with a mild strain to protect against marketable yield losses caused by its severe strains has been used widely in protected cropping [124,129]. The successful adoption of these control measures, combined with the current prevalence of its mild strains due to their use for cross protection purposes, has diminished PepMV’s importance over the last decade. However, the recent Israeli finding that, when preceded by ToBRFV infection, PepMV mild strain infections result in symptoms typical of its severe strains (Figure 3C), has raised renewed concerns over (i) the likelihood of its becoming more important again [124], and (ii) the wisdom of relying so much upon mild strain cross protection. After 2011, PepMV’s spread to further countries declined. This seems due to the improved health of seed multiplication crops resulting in distribution of largely uncontaminated commercial tomato seed internationally (Figure 4) [72]. 

### 7.2. Cucurbits

Among the world’s principal cucurbit crops, squash, zucchini (*Cucurbita pepo*) and pumpkin (*Cucurbita moschata* and *Cucurbita maxima*) were domesticated from their wild ancestors in the Americas in the pre-Columbian era, cucumber (*Cucumis sativus*) in India, and both watermelon (*Citrullus lanatus*) and gherkin (*Cucumis anguria*) in Africa; melons (*Cucumis melo*) were domesticated twice, once each in Africa and India/Southeast Asia [215,216]. Watermelon is the second most important fruit crop grown globally (https://www.quora.com/Which-fruit-is-the-most-popular-and-most-consumed-in-the-world). Cucumber and gherkin are the third most important vegetables, and the tenth most important are pumpkin, squash, zucchini and gourds (*Cucurbita* and *Lagenaria* spp. (https://www.statista.com/statistics/264065/global-production-of-vegetables-by-type). Although not ranked among the top ten fruits, melon is also a very important dietary component in many countries. An example of a recent major seed-borne virus disease epidemic of cucurbits is described below.

#### Cucumber Green Mottle Mosaic Disease

Cucumber green mottle mosaic virus (CGMMV; genus *Tobamovirus*, family, *Virgaviridae*) was amongst the earliest plant viruses described. This was in 1935 when it was found to cause a cucumber disease in glasshouse crops in England [217]. The disease symptoms were green mottling or mosaic of cucumber leaves and fruit surfaces [Figure 3D,E]. Subsequently, CGMMV was shown to cause disease in other vegetable cucurbits, fruit cucurbits and several cucurbit gourd species [39,130]. Between 1935 and 1985, CGMMV’s worldwide distribution expanded slowly; between 1986 and 2006, this expansion occurred more rapidly; and between 2007 and the present, it occurred very rapidly. It has now reached all continents apart from Antarctica [31,39]. Cucurbits are among the world’s most important vegetable and fruit crops (see above). CGMMV occurs in both protected cropping and open-field situations, its distribution is now global and the damage it causes is extensive. This means it is now the cause of one of the most serious cucurbit virus diseases threatening cucurbit fruit and vegetable production, food retail, and commercial breeding and seed companies [39]. Its characteristic foliage symptoms include mottle/mosaic (Figure 3D) that sometimes fades as the plant matures (especially in zucchini, squash and melon), or infection is mostly asymptomatic (pumpkin). As infected plants mature, watermelon foliage may become bleached, and in both cucumber and watermelon, infected plants may wilt, collapse and die prematurely. Fruit symptoms include skin mottle (cucumber) (Figure 3E), overall malformation, skin mottle and surface netting (melon) (Figure 3F), or internal flesh discoloration and necrosis (zucchini, squash). Watermelon fruits are the most severely affected, often being rendered unmarketable; their symptoms include overall malformation and internal sponginess, rotting and yellowing of the flesh, and necrotic streaking may develop in stems and peduncles (Figure 3G). Infected pumpkin fruits tend to be asymptomatic. Poor fruit quality due to CGMMV infection greatly diminishes marketable yields or renders fruit unmarketable [39,130,131,132,218]. Moreover, CGMMV also decreases gross cucurbit fruit yields [39,219], and the resulting yield losses in watermelon fruit can be >50% [132]. 

CGMMV has stable rod-shaped virions that remain infectious for long periods on contaminated surfaces and reach high concentrations in infected plants. Both of these factors favor its efficient transmission by contact when a healthy cucurbit plant comes into contact with an infected plant surface or with a contaminated soil [130]. Within protected cropping situations, it is efficiently transmitted by plant-to-plant contact, and by healthy plant contact with contaminated plant debris, soil, nutrient solutions or other contaminated surfaces, such as equipment, tools, hands and clothing used during pruning or trellising [39,133,134,220]. In the open field, it spreads to healthy plants by transplanting seedlings into contaminated soil, and contact with contaminated irrigation water, tractor tires, machinery, or other equipment moving through cucurbit crops [130,133,134,221]. CGMMV is also spread by bumblebee pollinators [135]. Moreover, due to contamination of the seed surface which allows seeds to remain infectious for very long periods, it is readily seed-borne in cucurbits. Planting contaminated seed stocks, or seedlings that became infected in seedling nurseries, both result in primary infection foci for further contact spread within cucurbit crops [39]. Fifteen alternative hosts of CGMMV belonging to nine different plant families have been reported from different continents [39]. The most thorough study was in Israel, where the wild alternative hosts found were: *Amaranthus graecizans*, *A. muricatus*, *A. blitoides* and *A. retroflexus* (*Amarthaceae*), *Ecballium elaterium* (*Cucurbitaceae*), *Chrozophora tinctoria* (*Euphorbiaceae*), *Moluccella laevis* (*Lamiaceae*) and *Withania somnifera* (*Solanacaeae*) [136]. Their roles in acting as virus reservoirs for CGMMV spread to cucurbit crops are yet to be studied [39]. 

Phylogenetic studies indicate an Asian origin for CGMMV, rather than a European origin, despite its initial detection under protected cropping in England [39]. The only cucurbit crops domesticated in Asia were cucumber and melon (see above) both being domesticated in the Indian subcontinent and melon also in Southeast Asia [215,216]. CGMMV likely first emerged as a disease of cultivated cucurbits in the Indian subcontinent by spillover of an ancestral virus from an unknown alternative host plant such as a wild cucurbit species, but phylogenetic studies with genomic sequences from the subcontinent are required to confirm this. In the last two decades, the rapid global increase in dispersal of CGMMV likely resulted from the practice of growing cucurbit seed crops in the Indian subcontinent and/or Southeast Asia without adequate control measures to limit virus spread, leading to seed crop infection, seed contamination and the unintended global dispersal of seed-borne infection by the international seed trade. Furthermore, this practice has likely also dispersed potentially more damaging CGMMV strains [39]. Evidence of the extent of CGMMV contamination in exported commercial cucurbit seed was provided when it was detected in cucurbit seed samples exported to Australia. CGMMV was detected in 4% (22/631) of samples [31]. It was found in seed samples of cucumber (8%; 8/102 samples), melon (3%; 13/393) and watermelon (2%; 1/57), and the CGMMV-contaminated seed lots came from seed crops previously grown in Europe, Asia, and North, Central and South America. This highlighted the need for seed testing as an important component of international border biosecurity [31]. Therefore, because of its recent increased importance and spread worldwide, CGMMV provides an example of a damaging major epidemic of a re-emerging virus disease that threatens food security. This threat has arisen from the practice of satisfying the international seed trade’s global all-year-round demand for supplies of cucurbit seed by multiplying seed crops in developing countries with warm climates where CGMMV already occurs. 

**Figure 5:**
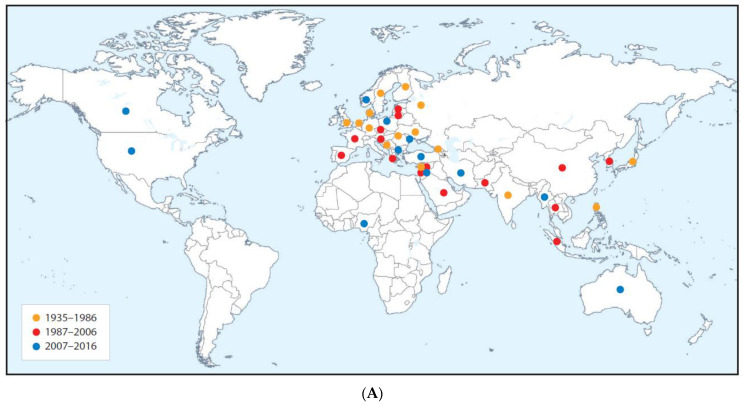

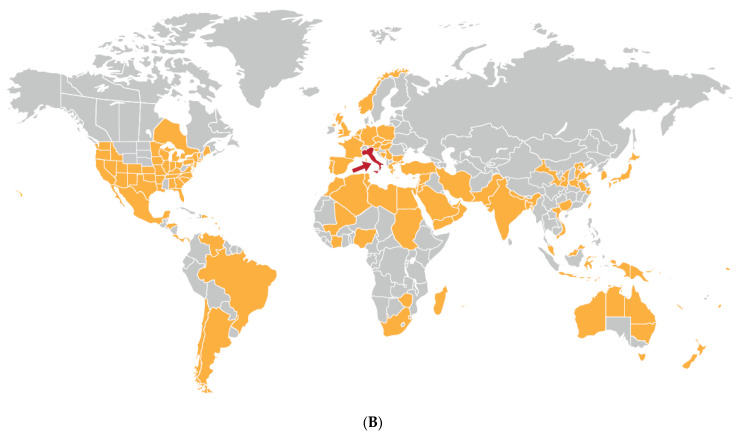
(**A**) Global spread of cucumber green mottle mosaic virus after its first detection in England in 1935. Colored circles show first detections within individual countries over three eras: yellow = 1935–1985; red = 1986–2006; blue = 2007–2016. This virus is readily seed-borne and was disseminated between continents, and across regions and countries, inadvertently, by the international cucurbit seed trade (figure credit @Volcani Center/Aviv Drombovsky). Figure modified from [39]. (**B**) Global spread of zucchini yellow mosaic virus after its first detection in Italy in 1973 (red arrow). During the following decade, it appeared in all continents with tropical, subtropical, Mediterranean, and temperate climates. It is seed-borne and was inadvertently disseminated between continents, and across regions and countries. This dissemination occurred mostly by the international cucurbit seed trade, but less often by infective aphid vectors blown in wind currents or international trade in infected fruit. The figure shows countries, and in some instances states within countries, reporting infection up to 2018 (figure credit @Washington State University/Naidu Rayapati). Figure from Supplementary data in [9].

Another seed-borne cucurbit virus, zucchini yellow mosaic virus (ZYMV; genus, *Potyvirus,* family, *Potyviridae*) is also the causal agent of a global virus disease epidemic that severely damages cucurbit crops in world regions with tropical, subtropical and Mediterranean climates. In cool temperate conditions, e.g., in Northern Europe, it is limited to protected cropping situations. [72,222,223,224,225]. Figure 5 illustrates the different phases of the increasingly rapid global dispersal of CGMMV since it was first found in England in 1935 (Figure 5A), and compares it with the even more rapid international dispersal of ZYMV since it was first found in Italy in 1973 (Figure 5B). 

## 8. Management

To prevent the spread of destructive virus disease pandemics or major epidemics to other continents, or other regions within the same continent, strict biosecurity and plant health regulations need to be enacted promptly and adopted rigorously. Such regulations need to prevent the viral pathogen, or virus complex, causing the disease from arriving in other continents or regions. This requires application of quarantine restrictions that prevent virus entry not only by the exporting country (pre-border), but also at land borders, seaports and airports (border) [19]. Success in preventing virus entry will be easier to achieve when the exporting country is geographically remote or separated by seas, mountain ranges or deserts than when national land borders have farmland on both sides. When attempts to prevent virus entry fail, quarantine regulations designed to eradicate or contain the incursion need to be applied promptly (post border) so that no further spread occurs [19]. However, in practice, when pre-border and border restrictions fail, successful eradication or containment is easier to achieve when the virus (or virus complex) spreads slowly (e.g., viruses with soil-borne vectors), its vector is absent, or it is adapted to thrive under different climatic conditions, especially temperature and rainfall variables [27,28]. Otherwise, the likelihood of successfully preventing or containing post entry spread may be limited. Therefore, rigorous application of pre-border and border restrictions that minimize the possibility that any incursions succeed in the first place is critical when it comes to preventing damaging plant virus diseases from being dispersed further afield. This is especially important when seed-borne viruses spread over long distances via international trade in unknowingly contaminated commercial seed stocks [30,31,39,203,204]. To avoid such inadvertent introductions, early detection of seed-borne virus contamination before seed is exported is critically important. Moreover, to avoid such dispersal, not only do seed crops need to be monitored for virus symptoms before harvest, and leaves from them sampled for virus testing, but also representative seed samples need to be virus tested after harvest. Furthermore, in case such testing is insufficiently rigorous, as a precaution, importing countries still need to test a representative subsample from every imported seed lot prior to its release [30,31,39,203,204]. 

When virus pandemics or epidemics are already taking place, integrated virus disease management programs need to be devised to ensure they are controlled efficiently. Devising these requires knowledge of the epidemiology of the causal virus(es) and their disease cycles [9,13,49,50]. Integrated disease management optimizes the effectiveness of virus disease control by combining suitable phytosanitary, cultural, chemical and host resistance control measures that work differently from each other and target distinct parts of the disease cycle. In protected cropping situations, biological control measures are included too, but these tend to be ineffective when deployed in the open field [9,13,49,50]. Every integrated virus disease management approach devised needs to be modified to take into consideration: (i) the characteristics and extent of the crop production system, which may range in from very large scale to smallholding or protected cropping in scale; (ii) the local climatic conditions; and (iii) any local societal or natural ecosystem restrictions that apply [9,13,49,50]. Thresh [12,13] focused on integrated disease management strategies suited to tropical conditions especially in developing countries, including ones tailored to virus diseases of perennial crops, and ones suitable for smallholder plantings. In the past, within developing countries with tropical climates, there was a tendency to rely solely or mainly on chemical control and breeding crops for virus resistance, whilst neglecting phytosanitary and cultural control tactics. Fortunately, this is now changing, with more comprehensive approaches being devised that include phytosanitary and cultural control measures, e.g., recommendations that include these types of control measures for managing diseases caused by geminiviruses [226]. 

To optimize the effectiveness of the control being achieved, whenever possible, new technologies should also be exploited. These include deploying remote sensing in association with precision agriculture to forecast: (i) epidemics on local, regional or continental scales via satellites; (ii) epidemics in individual crops via lightweight unmanned aerial vehicles; and (iii) precisely where to target control measures [227]. The latest innovations in information systems and predictive modelling should also be harnessed to predict the spread of virus pandemics and epidemics, and deliver advice over control options, such as Internet-based Decision Support Systems [227,228,229,230]. In addition, it is important to understand the full range of diversity of each virus (or viruses within virus complexes) that causes a plant virus disease pandemic or major epidemic. This is necessary so that the individual control measures included within the integrated disease management approach to be recommended can be adjusted to take virus diversity into account. Obtaining information about virus diversity requires studying a representative spectrum of virus isolates using a combination of traditional biological approaches, such as inoculation to differential plant hosts that distinguish diverse phenotypes [8], and genomic studies employing modern sequencing technologies, such as High-Throughput Sequencing (HTS), which reveal genomic variation [231,232,233]. A drawback to modern genomic studies is that the sequenced isolates often lack any biological data. Therefore, wherever possible, genomic studies should also include sequencing relevant isolates available within historical virus isolate collections so that linked biological data from the pre-sequencing era (disease symptoms, natural host range, virus transmission, epidemiology, etc.) can assist with the integrated disease management adjustment process [234].

In order to manage damaging global virus disease pandemics and major epidemics effectively, collaborative multidisciplinary research networks need to be organized. These require linkages between developed and developing country researchers, including plant virologists, entomologists, agronomists and plant breeders, along with modelers, statisticians, and socioeconomics experts [9,13,25]. An example of this is the international collaborative network providing solutions to two cassava virus disease pandemics in SSA [235]. 

## 9. Conclusions 

When staple food crops suffer large-scale losses from destructive virus disease pandemics or epidemics, this has major implications for people whose livelihoods depend directly or indirectly upon these crops. It can lead to dire circumstances of hunger and famine, especially in the world’s poorer countries. Similarly, damaging virus disease pandemics or epidemics afflicting non-staple food crops necessary for balanced nutrition have serious implications for human health. This review provides striking examples of the crop spoilage, losses in overall yield and produce quality, and hardship in the human population that arise. The virus disease examples described afflict six of the world’s most important staple food crops (maize, rice, wheat, potato, sweet potato, and banana), and five non-staple food crops that are critical for balanced human nutrition (citrus fruit, stone fruit, grain legumes, tomato, and cucurbits). Three of the six pandemic examples (MLND, RTD, and SPVD) are caused by distinct virus complexes and occur in open-field (arable) crops. The other three (BBTD, CTD, and PPD) are each caused by a single virus and affect plantation or orchard crops. By contrast, none of the seven major epidemic examples involve plantation or orchard crops. Four of these involve open-field crops (WYDD, WSMD, PTNRD, and FBNYD), and three occur both under protected cropping situations and in the open field (PepMD, TBRFD, and CGMMD). Most of the causal virus(es) of these 13 examples likely coevolved with the principal crop(s) they infect within their respective crop domestication center(s). However, proof of this requires phylogenetic analysis of sufficient virus isolate sequences from relevant crop domestication center(s). This is currently lacking in most instances—the four exceptions being for the causal viruses of PTNRD, RTD, BBTD, PPD. 

Most viruses responsible for the disease pandemics and epidemics described here became distributed between continents, albeit inadvertently, by long-distance dispersal of virus-contaminated seed or planting material. This occurred via either international trade in plants and plant products or germplasm introductions for plant breeding purposes. Such dispersal mostly involved virus-contaminated propagules of the affected crop rather than propagules of a related crop. In some instances, critically important vectors were distributed along with the virus-contaminated planting material, e.g., with BBTD and CTD. The causal viruses known to have spread by contaminated seed were MCMV, WSMV, PepMV, TBRFV and CGMMV, whereas those known to have spread by contaminated planting material were BBTV, CTV, PPV, PVY-R2, SPCSV and SPFMV. RTBV, RTSV, SCMV and SPMMV also likely spread via infected planting material, but MDMV via contaminated seed. The most important additional factors favoring virus disease pandemic or major epidemic development were:

(i) Introduction of vulnerable higher-yielding crop cultivars with MLND, WYDD, RTD, SPVD, BBTD, CTD, PPD and FBNYD;
(ii) Agricultural intensification to increase crop yields with MLND, WSMD, RTD, SPVD, BBTD, CTD, FBNYD, PepMD, TBRFD and CGMMD;
(iii) Virus recombination events resulting in more virulent virus variants with WYDD, RTD, PTNRD and TBRFD; and
(iv) Increased, or more efficient, vector populations with MLND, WSMD, BBTD and CTD.

As the world population continues to grow, the need to achieve overall global food security is becoming increasingly urgent. The escalating climate change-induced problems associated with controlling disastrous plant virus diseases threatening food crops constitute a crucially important issue for humankind to resolve. Therefore, achieving effective management of virus disease pandemics and major epidemics that harm not only staple food crops, but also crops essential for balanced human nutrition, is an extremely important goal. Achieving this goal requires international collaboration on a worldwide scale between researchers and other experts in relevant disciplines, extension personnel, plant biosecurity organizations, funding bodies, and policy makers. The objective is to develop a complete understanding of how each pathosystem behaves in different world regions, its disease cycle, its epidemic drivers and its overall epidemiology. This knowledge is essential for the development of biosecurity responses that prevent virus entry to a new region, or, where they fail to do so, eradicate or contain virus disease incursions after their arrival. It is also essential for optimizing the effectiveness of individual control measures. Finally, it is needed to devise integrated virus disease management and extension approaches directly suited to minimizing the deleterious impact of virus disease pandemics and epidemics upon food security and human health in a socially and environmentally responsible way. 

## Figures and Tables

**Table 1:** Examples of virus disease pandemics or major epidemics of critical importance for global food security.

Disease (Pandemic or Major Epidemic)	Continents or Regions Affected	Causal Agent(s)	Virus Genus	Vector(s)	Crop Diseased	Crop Domestication Center	Disease Impact	Virus(es) Origin(s)	Causes(s) of Appearance	Alternative Hosts	Factors Favoring Increased Importance/Distribution	Key Citations
Maize lethal necrosis (pandemic)	East and Central Africa, East Asia, Southeast Asia, North and South America, Europe	Virus complex: Maize chlorotic mottle virus (MCMV), plus sugarcane mosaic virus (SCMV), maize dwarf mosaic virus (MDMV) or wheat streak mosaic virus (WSMV)	*Machlomovirus*(MCMV), plus *Potyvirus* (SCMV, MDMV)) or *Trtimovirus* (WSMV)	Several beetle and thrips species (including the thrips *Frankliniella williamsi*) (MCMV). Several aphid species (SCMV, MDMV). Eriophyid mite (*Aceria tosichella*) (WSMV)	Maize (*Zea mays*)	North America (Mexico)	Widespread devastating yield losses. Food shortages (especially in East and Central Africa)	Probably all coevolved with the principal crop they each infect within each of its domestication centers: Mexico (MCMV, MDMV), Indian subcontinent and Southeast Asia (SCMV) Middle East (WSMV)	Ancestral viruses probably first spread by respective vectors to maize from wild maize ancestors (MCMV, MDMV), sugar cane ancestors (SCMV), or wheat ancestors (WSMV)	Sugar cane, Sorghum, millet, wheat, barley, and several pasture and weed grasses (MCMV). Several cereals and wild grasses (MDMV, SCMV, WSMV)	Seed-borne MCMV spread in contaminated maize seed resulting in mixed infections with locally occurring MDMV, SCMV or WSMV. Exacerbated by growing vulnerable maize cultivars, agricultural intensification to increase production and widespread occurrence of MCMVs *Frankliniella williamsi* vector	[42,71,72,73,74]
Wheat yellow dwarf disease (major epidemic)	Europe, Middle East, South, Central and East Asia, Southeast Asia, Oceania, North, Central and South America, North Africa, sub-Saharan Africa	Barley yellow dwarf virus (BYDV), and cereal yellow dwarf virus (CYDV). Distinct BYDV strains are PAV, MAV, RMV and SGV	*Luteovirus* (BYDV), *Polerovirus* (CYDV)	Aphids, especially *Rhopalosiphum padi* (PAV, CYDV), *R. maidis* (RMV), *Sitobion avenae* (MAV) and *Schizaphis graminium* (SGV)	Wheat (*Triticum aestivum*)	Middle East (Fertile crescent region)	Sporadic epidemics. Widespread yield losses	BYDV and CYDV both probably first originated in wheat’s Middle East domestication center. May have re-emerged separately in other world regions	Ancestral viruses probably first spread by aphid vectors from wild grasses to wheat May have done this several times	Barley, oats, rye, triticale, maize, rice and several pasture and weed grasses	Growing vulnerable wheat cultivars. Virus recombination generating virulent new variants	[4,72,75,76,77,78,79]
Wheat streak mosaic disease (major epidemic)	Europe, Middle East, Central and East Asia, Australasia, North and South America, North Africa, sub-Saharan Africa	Wheat streak mosaic virus	*Tritimovirus*	Leaf curl mite (*Aceria tosichella*)	Wheat (*Triticum aestivum*)	Middle East (Fertile crescent region)	Sporadic epidemics, severe yield losses, especially in Great Plains of North America.	Probably coevolved with wheat in its Middle East domestication center. May have re-emerged separately in other world regions	Ancestral virus probably first spread from wild wheat or grasses to wheat by *Aceria tosichella* vector. Likely occurred several times.	Barley, maize, oats, rye, sorghum, and some mostly annual grasses	Spread as seed-borne WSMV to other continents in contaminated wheat seed. Extended cropping periods and growing wheat under warm conditions that favor its mite vector	[4,72,79,80,81,82,83]
Rice tungro disease (pandemic)	Southeast Asia, East Asia (China) and the Indian subcontinent	Virus complex: rice tungro bacilliform virus (RTBV), and rice tungro spherical virus (RTSV)	*Tungrovirus* (RTBV)) and *Waikavirus* (RTSV)	Several leafhopper vector species transmit both viruses, *Nephotettix virescens* most efficient vector species	Asian rice (*Oryza sativa*)	East Asia (China)	Devastating yield losses, famine. Major deterrent to rice cultivation	Both viruses have the major phylogroups, Indian and Southeast Asian (RTBV), or Indian and Southeast Asian /East Asian (RTSV). Probably coevolved with rice separately within different parts of its domestication center	Ancestral RTBV and RTSV likely first spread by leafhopper vectors from infected wild rice or grasses to rice plantings	Wild rice and grass weeds often associated with rice paddies are alternative hosts of both viruses	Favored by agricultural intensification to increase production, growing vulnerable cultivars, and virus recombination generating more virulent variants	[4,13,14,61,62,84,85,86,87,88]
Potato necrotic ringspot disease (major epidemic)	All continents except Antarctic	Potato virus Y (PVY) necrogenic R2 variants	*Potyvirus*	Several aphid species, *Myzus persicae* most efficient vector species	Potato (*Solanum tuberosum*)	South America (Andean region (Peru, Ecuador)	R2-affected tubers unsaleable. Inability to manage effectively in many heathy seed potato schemes	PVY itself originally coevolved with potato in its Andean region domestication center	PVY ancestor spread by aphid vectors to potato from wild potato ancestors	Pepper, tomato, tobacco and many wild *Solanaceae* species	R2 arose in Europe by recombination between strains PVY^O^ and PVY^N^. It caused PTNRD and subtle or no foliage symptoms, and was more readily aphid transmissible. R2 spread globally in infected seed potato tubers. Largely unmanageable in many healthy seed tuber schemes	[40,58,89,90,91,92,93]
Sweet potato virus disease (pandemic)	Sub-Saharan Africa, North Africa, Middle East, Southeast Asia, East Asia, and North, Central and South America	Virus complex: Sweet potato chlorotic stunt virus (SPCSV) plus sweet potato feathery mottle virus (SPFMV), or sweet potato mild mottle virus (SPMMV)	*Crinivirus* (SPCSV), plus *Potyvirus* (SPFMV) or *Ipomovirus* (SPMMV)	Whiteflies *Bemisia tabaci* (SPCSV, SPMMV) *and Trialeurodes abutilonea* (SPCSV). Aphid species *Myzus persicae* and *Aphis gossypii* (SPFMV)	Sweet potato (*Ipomoea batatas*)	Occurred twice, separately in Central and South America	Devastating yield losses. Major deterrent to sweet potato cultivation	SPCSV and SPFMV probably coevolved with sweet potato in one of its two domestication centers in Central or South America. SPMMV spread to sweet potato from wild *Convolulaceae* hosts in East Africa	Ancestral viruses likely first spread by their respective vectors to sweet potato from its wild ancestors (SPCSV, SPFMV) or other wild alternative hosts (SPMMV)	SPCSV, SPFMV and SPMMV all infect wild *Convolvulaceae*: *Ipomoea* spp. (several species), *Hewittia sublobata* and *Lepistemon owariensis*	SPCSV and SPFMV spread globally in infected tuberous roots. Displacement of local sweet potato land races by vulnerable high-yielding cultivars. Spread favored by agricultural intensification to increase production	[72,94,95,96,97,98,99,100]
Banana bunchy top disease (pandemic)	Sub-Saharan and North Africa, Middle East (Iran), Indian subcontinent, Southeast Asia, East Asia, Oceania	Banana bunchy top virus	*Babuvirus*	Aphid (*Pentalonia nigronervosa*)	Banana, including plantain (*Musa* spp.)	Southeast Asia (especially Malaysia), Polynesia, Indian subcontinent	Devastating yield losses. Major deterrent to banana cultivation	Within banana’s wider domestication center, two major phylogroups diverged, the Pacific-Indian Oceans and Southeast Asian	Ancestral virus likely first spread by its aphid vector to banana from its wild ancestors or alternative hosts	*Musa paradisiaca M. textilis, and Ensete ventriculosum*	Wide-scale transportation of infected planting material of vulnerable cultivars to new geographic locations. Frequent new introductions of its *Pentalonia nigronervosa* vector. Agricultural intensification to increase production.	[38,63,64,72,101]
Citrus tristeza disease (pandemic)	South, Central and North America, Sub-Saharan and North Africa, Europe, Middle East, Indian subcontinent, East Asia, Southeast Asia, Oceania	Citrus tristeza virus	*Closterovirus*	Aphids. *Toxoptera citricida* most efficient vector. Less efficiently vectored by *A. gossypii, T. aurantii* and *A. spiraecola*	*Citrus* sp. Especially orange, lemon, mandarin, grapefruit, lime	Southeast Asia mainly, but also in the Indian subcontinent, East Asia and Melanesia	Devastating yield losses. Plants killed. Plantations abandoned	Co-evolved with *Citrus* species within the broader citrus domestication center (Southeast Asia, Indian subcontinent, East Asia and Melanesia)	Ancestral virus first spread by its aphid vectors to citrus from its wild citrus ancestors or related genera	Wild *Citrus* species and species in related genera, such as *Fortunella* and *Poncirus*.	Wide-scale transportation of CTV-infected and *Toxoptera citricida*-infested planting material to new geographic locations. Widespread growing of citrus trees derived from CTV-vulnerable cultivar scions grafted onto CTV-susceptible sour orange rootstocks.	[4,72,102,103,104,105,106,107,108,109,110]
Plum pox disease (pandemic)	Europe, Middle East, Indian subcontinent, East Asia, North Africa, South and North America	Plum pox virus	*Potyvirus*	Aphids. *Myzus persicae* most efficient vector	*Prunus* spp. Especially plum, peach, apricot, nectarine	China (peaches, nectarines). Europe, Asia, North America (plum, cherry)	Devastating losses in fruit quality, premature fruit drop and yield. Diminished orchard lifespan	Probably co-evolved with plum and cherry within its wider domestication center (central and eastern Europe and the Levant)	Ancestral virus spread by aphid vectors and grafting to plum and cherry from wild *Prunus* ancestors. Spread to peach and nectarine from infected *Prunus* after introduction to Europe and the Levant	Ornamental *Prunus* trees. Wild *Prunus* species	Spread by wide-scale transportation of PPV-infected *Prunus* planting material and germplasm to new geographic locations. Local spread by aphid vectors. Widespread adoption of vulnerable stone fruit cultivars	[4,14,60,72,111,112,113,114,115,116]
Faba bean necrotic yellows disease (major epidemic)	Europe (Spain), North Africa, Horn of Africa, Middle East and Arabia, Indian subcontinent (Pakistan)	Faba bean necrotic yellows virus	*Nanovirus*	Aphids. *Aphis craccivora, A. fabae and Acyrthosiphon pisum*.	Faba bean (*Vicia faba*)	Middle East	Sporadic epidemics. Devastating yield losses. Major deterrent to faba bean cultivation	Probably coevolved with faba bean (and other cultivated legumes) within faba bean’s domestication center	Ancestral virus probably spread by its aphid vectors to faba bean crops from other crop or wild legumes	Common bean, cowpea, chickpea, lentil and several wild and pasture legumes, and *Amaranthus* spp.	Growing vulnerable faba bean cultivars combined with agricultural intensification to increase production	[72,117,118,119,120]
Tomato brown rugose fruit disease (major epidemic)	Middle East, Europe, East Asia (China), North Africa (Egypt, Sudan), North (Mexico, USA) and South (Chile) America	Tomato brown rugose fruit virus	*Tobamovirus*	Contact transmission and by bee pollinators	Tomato (*Solanum lycopersicum*)	Middle East	Unmarketable fruit cause major losses. Recently spreading rapidly, especially in protected cropping	Uncertain. Possibly infected tomato in new encounter with indigenous virus in Middle East, or virus already coevolved with tomato beforehand but remained unnoticed until recently	Uncertain. Ancestral virus spread by contact to tomato from unknown host, or the virus itself was already present infecting tomatoes worldwide	Infects pepper. Possible alternative hosts include eggplant, petunia and the weed *Solanum nigrum* (natural infection yet to be confirmed)	Seed-borne international spread in contaminated tomato seed, and to a lesser extent in infected seedlings and fruit. Mutation or recombination event that broke resistance gene *Tm-2^2^*. Spread favored by intensive protected cropping procedures	[121,122,123,124]
Pepino mosaic disease (major epidemic)	South and North America, Europe, Middle East, Africa (Morocco, South Africa), East Asia (China)	Pepino mosaic virus	*Potexvirus*	Contact transmission and by bee pollinators	Tomato (*Solanum lycopersicum*)	Andean region of South America	Unmarketable fruit cause major losses.	Probably coevolved with, pepino, pepper and semi-domesticated tomato in the Andean region of South America	Ancestral virus spread by contact from wild tomato to pepino, pepper and tomato crops in the Andean region	Infects pepper and pepino crops, wild tomato species; and 18 weed species from seven different non-*Solanaceae* families in Spain	Seed-borne international spread in contaminated tomato seed, and to a lesser extent in infected seedlings and fruit. Spread favored by intensive protected cropping procedures	[7,72,124,125,126,127,128,129]
Cucumber green mottle mosaic disease (major epidemic)	All continents except Antarctic	Cucumber green mottle mosaic virus	*Tobamovirus*	Contact transmission and by bee pollinators	Fruit and vegetable cucurbits	Americas (squash, zucchini, pumpkin). Indian subcontinent (cucumber, melon). Africa (watermelon, melon, gherkin)	Diminished marketable yields or fruit unmarketable due to poor quality. Substantial gross yield losses.	Likely coevolved with cucumber and melon in Indian subcontinent domestication center. Spread from these two crops to squash, zucchini, gherkin watermelon, pumpkin	Ancestral virus probably spread by contact from wild cucumber or melon ancestor to cucumber and/or melon in Indian subcontinent	Infects cultivated gourds and wild species in nine families including *Amarthaceae, Cucurbitaceae, Euphorbiaceae, Lamiaceae and Solanacaeae*	Seed-borne international spread in contaminated cucurbit seed, and to a lesser extent in infected cucurbit seedlings and fruit. Spread favored by intensive protected cropping procedures	[39,72,130,131,132,133,134,135,136]

## Data Availability

Not applicable for reviews based entirely upon previously published information.
